# How the Working Memory with Distributed Executive Control Model Accounts for Task Switching and Dual-Task Coordination Costs

**DOI:** 10.5334/joc.138

**Published:** 2021-01-07

**Authors:** André Vandierendonck

**Affiliations:** 1 Department of Experimental Psychology, Ghent University, Belgium

**Keywords:** Working memory, Cognitive Control, Action

## Abstract

According to the working memory model with distributed executive control (WMDEC), working memory is not only used for temporary maintenance of information, but it also serves goal-directed action by maintaining task-related information. Such information may include the current action goal, the means selected to attain the goal, situational constraints, and interim processing results. A computational version of the WMDEC model was used to simulate human performance in a series of experiments that examined particular predictions regarding task switching costs, costs due to task and attention switching, to dual-task coordination in working memory tasks, and to experiments that required dual-task coordination of memorisation and task switching demands. The results of these simulations are reported and their implications for accounts of multi- and dual-tasking are discussed.

Human goal-directed action reconciles two opposing constraints: on the one hand an initiated action requires persistence in order to attain the intended goal, while on the other hand on-going action must be sufficiently flexible so as to allow rapid interruption or quick changes when these are called for. In order to better understand the mechanisms that underly actions that satisfy such opposing constraints, cognitive psychologists have developed specific methodologies that elicit such flexible goal-directed actions under controlled circumstances. To date, a broad range of procedures has been developed and studied, including procedures that vary from rapid switching among goals (task switching, for reviews see Kiesel et al., 2010; Vandierendonck, Liefooghe & Verbruggen, 2010) to performing two tasks simultaneously (dual-task coordination, e.g., Logan & Gordon, 2001; Pashler & Johnston, 1998), and many other variations.

In all these cases, two or more tasks (i.e., goal-directed streams of activity) partly or completely overlap in time while the degree to which the underlying goals are compatible across tasks may also vary. In terms of degree of overlap, a distinction can be made between procedures where, strictly speaking, the tasks do not overlap, procedures with a partial time overlap, and procedures with complete overlaps. When there is *no time overlap*, as in task switching procedures, task competition may still be involved due to overlaps between (memory) traces of the earlier task that conflict or compete with processes needed for the present task; this typically results in slower and more error-prone responding. In situations with *partial time overlap*, typically execution of a second task is started shortly after the execution of a first task. The PRP procedure is the example par excellence for this kind of situation (e.g., Pashler & Johnston, 1998). Although part of the task execution can be performed simultaneously, part of the processes can only be executed one by one which creates a bottleneck that results in delays on the execution of the second task (for a view that does not assume a structural bottleneck, see Meyer & Kieras, 1997b). In the third kind of situation, in which the two tasks *completely overlap*, several possibilities must be distinguished. First, two tasks have to be performed concurrently; for example, solving arithmetic sums orally while continuously categorising a tone as high or low by a manual response (e.g., Imbo & Vandierendonck, 2010). A second possibility is that during one task a series of several smaller but independent tasks have to be executed, as for example performing parity judgment on digits during the retention interval of a memory task (e.g., Barrouillet, Bernardin, Portrat, Vergauwe & Camos, 2007). A third possibility is the case of task interruption: one task starts first, but is interrupted to perform another one, after which the first task must be completed (e.g., Foroughi, Werner, McKendrick, Cades & Boehm-Davis, 2016).

In all such task settings, the tasks performed are rather independent from each other even though they may call on common processing mechanisms. A different kind of situation occurs when during the execution of one task another task is called for which is incompatible with the first task or which simply requires that the first task is stopped. Remembering a series of words while reading sentences (Hartsuiker & Barkhuysen, 2006) is an example of the former kind of situation. The latter kind of situation occurs in the stop-signal procedure: a task is being performed, but at any time a signal may occur to stop the first task as in the stop-signal task (e.g., Logan & Cowan, 1984, Verbruggen & Logan, 2008).

This large variation in situations that require control over the execution of multiple tasks has also stimulated a number of views on how the human mind performs these controls. A large range of theories has been proposed to account these kinds of control (e.g., Botvinick, Braver, Barch, Carter & Cohen, 2001; Logan & Gordon, 2001), but in the present article the focus is on views that consider executive control as part of working memory functioning. Working memory (WM) is involved to maintain at least interim results of processing as in the multicomponent working memory model (A. Baddeley, 1986, 2000; A. Baddeley, Hitch & Allen, 2020; A. D. Baddeley & Hitch, 1974; Hitch, 1978), but possibly also to maintain task goals, task sets and task progress. Miller, Galanter and Pribram (1960) were the first to propose the mediation of a working memory system to support goal-directed activities. WMDEC, the working memory model with distributed executive control (Vandierendonck, 2016, 2020) extends the conception of a multicomponent working memory system with the function of managing goal achievement by including a module that maintains task set information. The advantage of this combination of features is that the model not only addresses typical working memory phenomena, but also different kinds of multitasking such as task switching and dual-task coordination. The present paper examines these claims.

In what follows, first the model is briefly described, with a focus on a computational version. This computational version will be used to generate predictions for a number of published experiments on task switching and dual-task coordination.

## THE WMDEC MODEL

**Figure 1:**
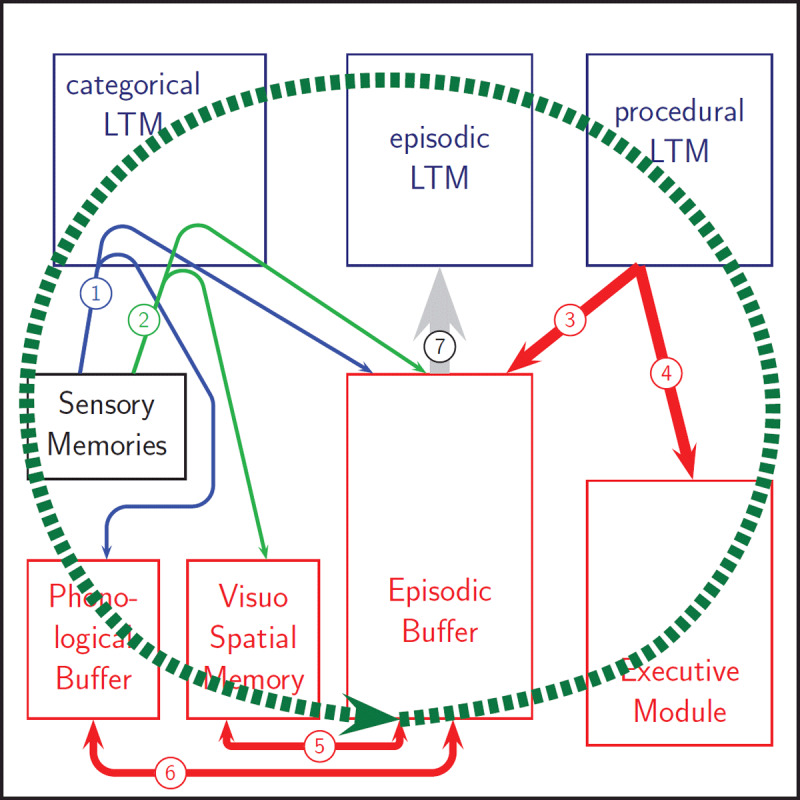
Schematic overview of the WMDEC model. Working memory consists of four modules (shown in red in the lower part of the figure), namely the phonological buffer, the visuospatial module, the episodic buffer and the executive module. The figure also shows the sensory memory systems (on the left) and the long-term modules in the top part (in blue). Environmental information is shortly kept in sensory memory. The procedural loop (big circular arrow shown as a thick dashed green line) continuously compares condition-action rules stored in procedural long-term memory to sensory and working memory modules. One of the matching rules is selected for execution (routes 3 and 4). Sensory events are interpreted by consulting categorical long-term memory and can be instantiated in the episodic buffer and the modality-specific systems via routes 1 and 2. The episodic buffer also interacts with these modality-specific systems via routes 5 and 6 and over time the buffer contents may flow over into episodic long-term memory via route 7.

Like the multi-component working memory model of Baddeley and colleagues, the WMDEC model consists of a multi-modal episodic buffer assisted by two modality-specific systems, namely the phonological buffer (and loop) and the visuospatial system. However, unlike Baddeley’s multicomponent model, the present model does not include a central executive, but instead it contains an executive memory that is dedicated to the storage of task-set-related information. ***Figure 1*** displays the structure of the model and its interrelation with long-term and sensory memory. The model endorses four basic principles, namely (1) WM provides storage space to support planned actions and goal attainment; (2) WM includes an inner speech mechanism for self-instruction and rehearsal; (3) Control processes are an inherent part of WM and are embedded in learned rules; (4) WM is a versatile and flexible system that allows different encoding formats at variable levels of generality.

The WMDEC model was proposed as an alternative to the multicomponent model of Baddeley and colleagues (A. Baddeley et al., 2020; A. D. Baddeley & Hitch, 1974; A. D. Baddeley & Logie, 1999) in which the *central executive* was replaced by a set of low-level control processes. By doing so it was also an alternative to other models calling on a central executive (e.g., N. Cowan, 1999; N. Cowan, Morey & Naveh-Benjamin, 2020) and runs parallel to Logie’s proposal to retire the central executive (Logie, 2016). The model’s mechanism for low-level control processes provides an alternative account for what is called activated procedural LTM in Oberauer’s WM model (Oberauer, 2009, 2020) and the model yields a process-driven account for the notion of cognitive load which is prominent in the WM model of Barrouillet and Camos (Barrouillet, Bernardin & Camos, 2004; Barrouillet & Camos, 2020). In other words, the model is in competition with recent major conceptualisations of working memory and has its roots in the ideas of Miller et al. (1960) and the modal short-term/working memory model of Atkinson and Shiffrin (Atkinson & Shiffrin, 1968; Shiffrin & Atkinson, 1969).

The studies reported in the present article are all based on a computational implementation of the WMDEC model. Computational versions of WM models have been around for several years now, but most of these focus on a particular aspect of WM functioning. In this vein, several models of the phonological loop have been proposed, such as the model of Burgess and Hitch (1999, 2006) and the primacy model of Page and Norris (1998). Likewise, the serial order in a box models by Farrell and Lewandowsky (2002) and its extension to complex span situations (Oberauer, Lewandowsky, Farrell, Jarrold & Greaves, 2012, address particular aspects of Oberauer (2009)’s design for working memory. Also a few computational versions of the Time Based Resource Sharing model (Barrouillet et al., 2004; Barrouillet & Camos, 2020) have been attempted, one by Oberauer and Lewandowsky (2011) and another one by Glavan and Houpt (2019). In the book on models of working memory by Miyake and Shah (1999) several chapters address computational approaches to working memory, among others a model based on ACT-R by Lovett Reder and Lebiere (1999) which focuses on working memory and its relation to complex cognitive activities. Kieras, Meyer, Mueller and Seymour (1999) presented the EPIC model as an approach to WM (Meyer & Kieras, 1997a, 1997b). While all these modelling attempts tend to focus on particular aspects of WM and the scope of some of these models is quite broad, the present implementation aims to integrate WM functioning and control of task planning and execution in a single model with specific mechanisms to address goal-directed activities.

Because an extensive explanation of the general structure and functioning of the WMDEC model in a variety of situations has been already published (for more details, the interested reader is referred to Vandierendonck, 2016, 2020), the description of the model will focus on the implementation of the computational version of the model. First, it is important to stress that the qualification ‘*distributed executive control*’ is used to make a distinction with WM models that assume a central agent, such as a central executive to account for executive control. In WMDEC, executive control is not covered by a central agent, but is spread over processes that achieve such control in specific situations. In other words, executive control is *distributed* over lower-level processes that have been acquired via associative learning (e.g., Abrahamse, Braem, Notebaert & Verguts, 2016).

In what follows, the operation and implementation of the model will be explained in detail and sufficient context will be provided to give an insight in the principles and their underlying rationale. The operation of the model is based on a continuous interaction of the environment and the memory system via perception and detection of environmental changes and via outputs from the system that may alter the environment. This operation runs over time, but at any moment the different WM modules are operating simultaneously (in parallel). Because the computer program running the model performs every step in sequence, parallelism is achieved by partitioning time into short slices so that everything that happens in such a time slice is considered to be occurring at the same time. In order to avoid leaking of events from one time period to the next, all the actions of the different modules are programmed to occur in a fixed order within the time slices. This was achieved by defining a *procedural loop* that completes one such time period at a time, such that every run over all the processes within the time interval constitutes one cycle of this loop. For convenience the duration of a cycle was set at 10 ms.^1^ In what follows, I explain the implementation of each of the modules of the model before addressing the dynamics of model operation.

## MODEL STRUCTURE

### Declarative Long-term Memory

Declarative or categorical LTM (dLTM) is represented as an associative network in which the elements are connected to each other by means of labeled links. A distinction is made between superordinate, subordinate, property, opposition and sequence links. More details about the entities in dLTM, their links and their meaning is provided in ***Appendix A***.

On the one hand, dLTM represents information about the environment which in the computational implementation is limited to these objects and events that are needed for the simulation. In the experiments, the digits 1–9 (D1–D9),^2^ the alphabet (WA–WZ), cues signalling which task to perform (e.g., CMAG for a cue to the magnitude judgment task), etc. are included. On the other hand, dLTM also contains symbols that refer to ‘internal events and objects’, such as GOAL, TSKSET (task set), CHUNK, etc.

Every entity in dLTM is a node in a network where each node has a name, and one or more relations (labeled links) to other nodes. If the node is a verbal element it also has information about the phonological code (expressed as the number of syllables). Each node also has a strength (0–1) which on principle can change by learning and experience, but this aspect is not part of the presently used computational version. Strength expresses the likelihood that access to the entry succeeds at a particular moment. In the present implementation all nodes are assigned a strength of 0.50; this value ensures that all nodes are directly accessible on the rationale that the set of knowledge that is used in the simulations is permanently accessible. The strength parameter is included to allow variation of the likelihood of access in future versions.

The complete list of specifications for each node is uploaded by the program at the start of a run. The uploading function also checks the incoming information for consistency and adds some of the missing links (e.g., if element x is a subordinate of y, then y must also be superordinate to x).

### Procedural Long-term Memory

Procedural LTM contains procedural knowledge which is essentially a collection of production rules also known as condition-action rules. Because the concept of production rule plays a key role in the computational version of WMDEC, first the notion of production rule is explained. By means of some examples, it is made clear how these rules were implemented.

A production rule consists of a condition that if matched by a certain state of affairs triggers execution of the action part of the rule. It is defined as ‘**if** *condition* **then** *action*’, where *condition* is an expression which is true or false, and *action* refers to a function or procedure. A condition is either a simple statement that is true or false or it is a combination of several such statements connected by the **and** operator; such an elementary statement can be negated, reversing its truth value. A simple example of a condition-action rule is

**if** X is a new object in sensory memory **and** X does not exist in the episodic buffer **then** create X.

In this example, the action specifies that a representation of the object X is to be created (in the episodic buffer).

When the condition of such a rule is true, the rule is said to match the current situation. The condition is true only if the entire expression in the condition is true. For the example this implies that if X is a new object in sensory memory but it does already exist in the episodic buffer, the condition expression is false and the rule does not match.

For the implementation of a model, a larger degree of detail than present in the example above is needed. The rule used in the present implementation actually was the following

**if** newcnt(ISM) == 1 **and** X = isnew(ISM,1) **and** !ltmhas(X,TARGET) **and**

ltmsup(X,EXTL) **and** !ineb(X,ANY,ANY,NOLOC,0) **and**

!ineb(ANY,CUE,ON,NOLOC,0)

**then** mkinst(X,ANY,NOLOC)

This rule uses a number of comparison functions that are applied by the computer program to assess the their truth value. These functions are documented in ***Appendix B***, which also defines the syntax for the productions in the present implementation. These functions cover checks in all the modules of the WM system. The rule shown here, first checks wether there is one new element (newcnt) represented in Iconic Sensory Memory (ISM), and if so (isnew) assigns its name to the variable X, and then consults dLTM (ltmhas) to see whether it has the property of memory TARGET and if it has not, further checks dLTM to assess (ltmsup) whether the value of X is an external event or object and if so checks whether it not already has a representation in the Episodic Buffer (ineb) or whether it has been represented in EB as a cue. If all these conditions are true, the action mkinst (‘make instance’) is executed. All these checks are needed to avoid that the perceived object is something that does not already exist or does not deserve to be attended to and to be represented in WM.

The complete set of production rules represents the procedural knowledge which is necessary in the experiments simulated in the present article. This set of rules is uploaded into pLTM at the start of a run. Over the run of the model, changes to the set of rules do occur on the basis of experience and learning (the learn function). There are two important ways in which these rules change. The first of these is based on changes made to the strength of the rules. At the start, each rule has a strength *s*. Each rule that has been selected for execution during a trial of an experiment or during a particular episode will be strengthened or weakened depending on wether the rule application contributed to a positive outcome (e.g., correct response) or to a negative outcome (incorrect response), according to the following equation

1 {s_{t + 1}} = \left\{ {\begin{array}{*{20}{l}}
{{s_t} + (1 - {s_t})\eta }&{{\rm{if\ positive\ outcome}}}\\
{{s_t}(1 - \eta)}&{{\rm{if\ negative\ outcome}}}
\end{array}} \right.

where *s_t_* is the production strength at time *t* and *η* is the learning parameter which has the value 0.00008 (see ***Table 1*** for all the model parameters and their value in the present application of the model). A second way in which production rules can change consists of the creation of new rules on the basis of experience. More particularly, when a stimulus *S* was followed by a response *R* in a particular trial or episode and the outcome was positive, a rule of the following form is created:

**if** V = domts() **and** ltmsup(V,TASKTS) **and** ineb(ANY,OBJECT,ON,NOLOC,1) == S **and** gamsget(STATUS) != DONE

**then** respond(R).

**Table 1:** Model parameters and their standard settings.


NAME	EXPLANATION	VALUE	LOW	HIGH

EB and EM parameters				

*α*	Instance activation growth rate	0.02	0.005	0.035

*β*	Lateral inhibition rate	0.99	0.985	0.995

*φ*	Initial activation of new instance	0.25	0.20	0.30

*τ*	Inhibition rate of rejected instances	0.75	0.65	0.85

	EB maximal activation capacity	2.90		

	EM maximal activation capacity	5.0		

PL and VSM parameters				

*δ*	Phonological decay rate	0.989		

*ρ*	Activation growth rate in rehearsal and revival	0.10		

*σ*	Visual decay rate	0.99		

LTM parameters				

*η*	Rule learning rate (pLTM)	0.00008		

*Κ*	Consolidation rate (eLTM)	0.001		

Attention and Motor parameters				

*θ*	Response threshold (neutral)	0.50	0.40	0.60

*λ*	Standard deviation of gaussian distribution for response production	0.01		

*ζ*	Mean of gaussian noise distribution for goal-directed response production	0.015	0.010	0.020


This rule states that if V is the dominant task set and it is known as such (in dLTM) and S exists in the episodic buffer and motor information is still available, then produce R. In this rule ‘S’ and ‘R’ refer to the respective names of the stimulus and the response involved; the rule is assigned a starting strength of 0.05 which can increase or decrease on later occasions depending on the success of the new rule. The rule is also assigned a duration of 5, because apart from a strength, every rule also has a duration, which specifies the number of cycles minimally needed to complete the action. However, when the object of the action is busy to another rule, the action will only start as soon as the other rule finishes and continue for the number of cycles specified.

### Sensory Memories

Objects or events in the environment become available in the iconic sensory memory (ISM) via visual perception or in the echoic sensory memory (ESM) via auditory perception. Visual objects or events occur in a spatial location. Because no fine-grained spatial location is needed for the presently reported applications of the model, a 9 by 9 location matrix was used to represent objects/events in a two-dimensional space as well in WM as in ISM. Each cell of this array is either empty or contains one single object or event that is characterised by a symbol name, a flag, a colour and a trace strength. The flag can be NEW (new non-processed element), CHANGE (the previous element is replaced), FADE (after some time the element starts to fade out), GONE (the object is no longer present). An empty cell is flagged as EMPTY. This design implies that visual objects or events can occur simultaneously and can be simultaneously maintained in ISM as long as they are in different locations.

When objects are simultaneously present, they will in general not be processed at the same time. A first reason is that for further processing, these objects/events must be matched by a production rule. Once a rule is selected for processing one such object, it will not be available for processing other objects as only one rule at a time can be selected for execution. So the simultaneously present objects will in general be encoded in WM one by one. However, with locations spread out in space, not all objects may fall within the fixation area and then a saccade may be needed to bring some of the other objects into focus. This will take some time and will contribute to a further spread over time (cf. infra).

For auditive perception, localisation of the source is more difficult to achieve. In principle the model assumes that roughly three locations can be distinguished (left, centre, or right), but in the present implementation the number of locations was limited to one. Echoic elements also have a symbol name (needed to access dLTM), a flag and a strength. Because of the sequential nature of auditory inputs, the flags are BUSY (at the start of the auditory stream when no identification is yet possible), NEW and CHANGE (when part of the stream has passed), FADE at the end of the stream and GONE (1.5 second after the end of the stream).

### Episodic Buffer

When objects present in sensory memory are selected for further processing, they will be elected for representation in the Episodic Buffer (EB). In that case the information present in dLTM is used to create an instance (a representation combining dLTM and input information) in the EB. Depending on the case at hand and the information already present in WM, the newly created instance may have connections to the Phonological Loop and/or the Visuospatial Module. Because the EB is one of the core modules of working memory, the traces created in this memory module are rich in information. Each such trace has apart from a symbol name (corresponding to an entry in dLTM), a type (GOAL, COORD, CUE, OBJECT, CLASS, RESPONSE, CHUNK, or BIND), a spatial coordinate (if it is represented as an object in space), a link to dLTM, an episode identifier (corresponding to the episode in which the trace was created or re-created), a time stamp (the number of the cycle in which it was created or its creation will be complete), a busy flag (a reference to the production rule acting on the element or 0), an access indicator (OPEN or CLOSED, depending on whether it is accessible or not at the present time; for the rationale behind this, cf. infra), a flag (EXT when an external event to which it may refer is present, INT when such an external event is not present, or DONE when this trace is no longer needed), a mark (ON for active, OFF for no longer active or TBR if a recall is likely needed), an activation level or strength, a pointer to a trace in the visuospatial module (or 0), a pointer to a trace in the phonological loop (or 0), a pointer to a trace in Episodic LTM (or 0), and a recall status if applicable. Furthermore, if the trace is for a chunk, it will contain links to the composite traces and to dLTM, or if it is a bind it will also contain links to the EB elements that are bound together. Most of these properties provide points of comparison for production rules; for example, some production rules will only match if the instance is marked as TBR, other rules will only match if the instance has a particular type (e.g., GOAL or BIND), etc.

Creation of an instance occupies several cycles. It depends on whether the instance is new or is a re-creation of an older no longer used trace. The duration to set up a new instance is

2 d = 20 + 4r

where *d* is the number of cycles and *r ∈ N*(0,1) is a gaussian random number. At the start the activation level of the instance is *φ* (default 0.25). Until the creation is completed, on each cycle this degree of activation is increased according to the following rule

3 {a_{t + 1}} = {a_t} + \,\,(1 - {a_t})\,\alpha

where *a_t_* is the activation at time *t* and *α* is the growth rate parameter which also applies during refreshment of EB traces (cf. infra). In case the newly created instance is a competitor of a trace (according to the isop relation in dLTM), the two traces will inhibit each other (mutual lateral inhibition), which applies on every cycle until the creation is complete. Such inhibition occurs on the basis of the following rule

4 {a_{t + 1}} = \,\,{a_t}\beta

where *β* is the inhibition parameter (default 0.99). Once created, an instance does not decay, but because of the limited capacity of EB, competition may result in loss of information. EB has a capacity limit *C* (default 2.9) which is exceeded whenever the total amount of activations in EB is larger than *C*. Given item activations *a_i_*, the total activation is {\sum\nolimits _i}{a_i} and the ratio to available capacity is f = {\textstyle{C \over {{\sum _i}{a_i}}}}. When the value of *f* is smaller than 1, a correction occurs in two steps. First, decrease of activation is limited to the elements that were created in an earlier episode (or trial) or are flagged as DONE; their activations are multiplied by the fraction: *a_i_ f*. Next, if capacity is still exceeded, the fraction is recalculated and applied to all elements.

Finally, when instances are no longer needed, as for instance when they are combined to form a chunk, they are inhibited by applying the rule

5 {a_{t + 1}} = \,\,{a_t}\tau

where *τ* is the inhibition rate (default 0.75). This rate is also used in other contexts where inhibition occurs (cf. infra).

Loss of information from EB can be counteracted by attentional refreshment (cf. Johnson, 1992; Johnson et al., 2005). Refreshment occurs only for EB traces that are marked TBR (to-be-recalled) and is controlled by the memory task set according to the DREF parameter which specifies the schedule to be followed: all traces, only the oldest one, two or three or only the most recent one, two or three traces. At any one time only one trace can be refreshed which involves an incrementation of the activation according to Equation 3.

### Phonological Loop

The Phonological Loop (PL) is a storage medium that supports rehearsal. When particular events or objects have to be retained for later recall, and rehearsal is active, then a phonological trace is made that is linked to the EB. Such a trace consists of a symbol name, a link to an EB element, an access indicator (OPEN or CLOSED), the number of syllables of the trace, activation level (between 0 and 1), and a link to the next element in the loop. As the list of traces forms a loop, the last trace points to the first; when the loop contains only one element, it points to itself. At the time of creation, the trace is assigned a value of 1.0. Over time, the PL contents decay at a rate specified by the parameter *δ* according to the following rule

6 {a_{t + 1}} = {a_t}\delta

where *a_t_* is the activation of the PL element at time t. In the present implementation *δ* = 0.989 to ensure that it takes about 4 seconds to decay from 1 to 0, yielding a half-life time of 2 seconds. Rehearsal, if applied, is an automatic mechanism that addresses each element in the loop in turn and reactivates this element according to Equation 7

7 {a_{t + 1}} = \,\,{a_t} + \,\,(1 - {a_t})\,\rho

where *ρ* is the rehearsal strengthening rate. The process of rehearsing the element has a duration of *d* cycles:

8 d = \,\,0.9\sqrt n {t_s} + \,\,r

where *n* is the number of syllables of the phonological element, *t_s_* is the duration of a syllable (50 cycles presently) and *r* is a gaussian random number *∈ N*(0,1) rounded to an integer value.

### Visuospatial Memory

The Visuospatial Memory (VSM) system keeps visual and spatial representations active in a way comparable to what the phonological loop does for verbal and auditory materials. For storage, it consists of an array (9 by 9 in the present implementation) in which representations are bound to a location in two-dimensional space. The locations are either EMPTY or contain a representation with a symbol name that allows access to dLTM, a type (word or other symbol or a shape), a flag (EXT, INT or DONE depending on the status of the corresponding element in ISM), the colour of the represented object, an activation level, a link to a corresponding unit in the episodic buffer, the two-dimensional coordinates of its position, a pointer to the next element of an ordinal sequence, and an access indication (OPEN or CLOSED). The contents of this memory decay over time at a rate *σ* (0.99 in the present implementation) as specified in Eq.9

9 {a_{t + 1}} = \,\,{a_t}\sigma

where *a_t_* is the activation of the element at time step *t*. This decay can be counteracted by revival (similar to rehearsal but for visual images) and this proceeds in the same way as specified in Eq.7 for rehearsal with the provision that a single revival duration is given in

10 d = \,\,25 + r

where *d* is the duration of the revival and *r* is a Gaussian random number *∈ N*(0,1) rounded to an integer value.

### Executive Memory

The Executive Memory (EM) system maintains all information that is relevant to goal achievement. In the context of a memorisation task, for example, this consists of maintaining the to-be-recalled elements in memory, and by organising recall when this is requested. In the context of other kinds of tasks, this involves keeping track of the task constraints (e.g., respond only to red objects), keeping track of the task settings and selecting an appropriate route to responding. In both kinds of situations, EM will maintain a task set which may include particular actions (e.g., memorisation, recall, or recognition in a memorisation context), particular parameter settings (such as required response modality, response control, etc.) and category-response mappings. A task set can best be conceptualised as a schema maintained in LTM (cf. Abelson, 1981; Graesser & Nakamura, 1982) based on prior experience that may contain a number of default values for its constituents.

In the present implementation a task set is a structure that has a name (to access dLTM), an activation level, an access indicator (OPEN/CLOSED), a time stamp (cycle of creation), a busy indicator (ON or OFF; if ON the task set is already involved in an operation), a mode (applicable for tasks sets with several actions, such as the memorisation task set), a list of uploaded task-set parameters, a list of uploaded mappings, and a list of uploaded actions, if any. Parameter settings are simply key-value pairs with a particular strength. Mappings are simple conditional rules (a simple condition and a response), they have a symbol name and a strength. Task actions are more complex structures because they must keep track of progress with the action; they have a symbol name, an access indicator (AWAKE or SLEEP) depending on the mode setting in the task set, an activity status (EMPTY, ON, HOLD, FREE or DONE), an indicator of the object type to which the action applies (WORD or SHAPE), an indicator of the task (sequential or simultaneous), for each of PL, VS, and EB, a pointer to the current target in the module and the number of successive access failures for this target, current reference age of the object, number of matches, number of detected to-be-recalled elements, action count, and cycle at which the action completes. Many of these indicators are specific for retrieval-related actions.

EM has a limited capacity (*C* = 5 in the present implementation; this value includes the activation of the active task sets and all their components). If the capacity is exceeded, activations are downwards adapted in the same way and according to the same formula as in EB.

#### Episodic Long-term Memory

Traces in the EB can flow over into the episodic Long-term Memory system (eLTM). In the computational version this is limited to EB instances that are refreshed. Traces in eLTM have a symbol name, a pointer to dLTM, an episode indicator, a time stamp, a sequential position indicator within the episode, a strength and possibly a spatial location. Refreshed EB traces become consolidated in episodic LTM; this occurs according to the following formula

11 {a_{t + 1}} = \,\,{a_t} + \,\,(1 - {a_t})\,\kappa

where *a_t_* is the degree of activation in eLTM and *κ* is the consolidation rate (0.001 in the present version). This is updated once per cycle but only for a trace of EB that is being refreshed.

### OPERATIONAL DYNAMICS

It was already pointed out that parallel processing is approximated by handling simultaneous actions in a fixed order in short spaces of time and that all the things that must happen are performed in a fixed order to avoid any anomalies or any leaking of information from one time period to the next. In each cycle of the procedural loop the following actions are performed in the order listed:

1. If a response is ready for output, this is preserved to be send out after all other actions within the cycle are performed.
2. Next, the activation levels of the traces in the sensory memories, and in the phonological loop and visuospatial working memory are decreased (decay), overflow of capacity limits in the episodic buffer and executive memory are checked and activations are adapted if necessary.
3. If appropriate, a rehearsal step is initiated in the phonological loop.
4. Likewise, a revival step is initiated in VSM.
5. If refreshment is active, the refreshed EB trace is consolidated in episodic LTM.
6. Response tendencies that are present are increased on the basis of a random walk (cf. infra).
7. Access flags in all memory modules are set. The rationale for this is as follows: each memory trace in each of the WM modules has a degree of activation which determines the likelihood of accessing this trace. This depends on a randomly generated value. If this value has to be selected at each attempt of access within the cycle, access would vary within the same cycle. Such unwanted and unwarranted variability is avoided by fixing the access within the cycle: a uniformly distributed random value between 0 and 1 is obtained and if a function of the random value is larger than the degree of activation, access is OPEN, otherwise it is CLOSED. In the phonological loop, if *r* is the uniform random value, the function is (0.6*r*)^2^ so as to always allow access to traces that haven’t been rehearsed for 2 seconds. In EB, EM and VSM, a step function is used: activations above 0.5 are always accessible; below this threshold, access is a linearly decreasing function of *r*, namely 0.5*r*.
8. Set spatial fixation and focus (more detail later).
9. Adapt the contents of the sensory memories taking into account any changes in the environment.
10. Adapt EB contents to reflect changes in sensory registers (e.g., when an EB trace is based on an element in sensory memory it is flagged as EXT, but if the sensory memory no longer exists, this flag must be changed to INT).
11. Compare all production rules (except the ones active at this moment) to the current state of the system and if there are any matches, select one new production rule for execution.
12. If a response is ready, interrupt the loop so as to pass on the response to the environment for proper follow-up; after this the procedural loop will be called from the environment to continue its actions.

From the explanations thus far, it should be clear that besides memory modules also other systems must be included to achieve a complete implementation. One issue concerns interactions with the environment and the other concerns general attention and responding.

#### Interaction with environment

The current state of the environment is defined at the start of a run and consists of a list of events that are present from time *t* with a particular duration *d* until and including *t + d –* 1; the event has a symbol name, a colour, and occurs at a particular position in space (if it is a visual event). As an example, ***Table 2*** shows the sequence of events in a task switching experiment: a fixation cross, then a task cue, a stimulus or target and an empty interval. The fixation cross is present for 50 cycles. The next two events have a variable but limited duration: they are present until the response is given or until 300 cycles have elapsed, whichever comes first. So the cue is planned to be present for 390 cycles (300 + 90 cycles for the cue-target interval); the target is planned to be present for 300 cycles. When the response occurs, control comes back to the environment to assess the effects of the response: in this case it is correct and the durations and endings of the cue and the target are registered (shown in the bottom panel of the table) and the start and ending of the empty event are shifted to occur immediately after the response. After these changes, control goes back to the procedural loop.

**Table 2:** Interaction with environment via definition and adaptation of states. Example from a task-switching context.


NAME	COLOUR	MODALITY	POSITION	START	DURATION	END

CROSS	BLACK	VSP	4,4	0	50	49

CMAG	BLACK	VSP	4,3	50	390	439

D9	BLACK	VSP	4,5	140	300	439

EMPTY		VSP	4,5	440	50	489

Adaptations after response						

CMAG	BLACK	VSP	4,3	50	181	231

D9	BLACK	VSP	4,5	140	91	231

EMPTY	BLACK	VSP	4,5	232	50	281


*Note*. All the attributes of the environmental events are shown. The position is only applicable for events in the visuospatial modality (VSP) and gives the position in the 9 by 9 matrix used for spatial locations.

#### Interactions with attention and motor events

In order to make the implementation work properly, it is necessary to include some program code that ensures that WM is properly embedded with respect to attentional and motor issues. It was already mentioned that events in the environment may occur at different locations in space. As far as visual input is concerned, it is well known that at any time only a part of space is perceived with sufficient acuity for further processing. In the present implementation, it is assumed that at any time the visual *fixation point* corresponds to one of the ‘cells’ of the 9 by 9 visuospatial matrix and that the fixated area corresponds to only that cell and its direct neighbours. Therefore if an event occurs outside this area, a saccade is needed towards the critical area. At the start of a run, the fixation position is selected at random or when the fixation has lasted for 20 + 3*r* cycles, where *r ∈ N*(0, 1) is a gaussian random number, the fixation drifts to a randomly selected position 1 cell away in any direction. During the saccade (3–6 cycles, depending on the distance) no visual inputs can be processed. All the settings needed for these operations are maintained in the spatial part of the ‘General Attention and Motor System’, namely the status (fixation, preparing random saccade, preparing stimulus-based saccade, executing saccade), the current fixation point, the next fixation point, the duration of the saccade in progress and the distance of the saccade.

This general system contains also the settings needed to *control motor operations*. In tasks requiring a motor response, execution of the response is the end of a process in which at some point a response is selected after which the motor system is prepared to emit this response. At about the same time, a response (the same or another one) may have been automatically triggered on the basis of a previous event or on the basis of an acquired automatism. The competition between such response tendencies is included in the model on the basis of a random walk process roughly similar to the drift diffusion model of Ratcliff (1978). Once the response process is initiated, in every cycle the activation of the response(s) is increased; the response of which the activation exceeds the response threshold first is the one executed and the cycle at which this occurs determines the response time. Assuming two response tendencies (one from an intentional stream, i.e., initiated by the *execute* action, and one from an automatic stream, i.e., initiated by the *do* action), their activation is changed every cycle by adding *λr + ζ_k_*, where *r ∈ N*(0, 1) is a gaussian random number, *ζ*_0_ (intentional stream) is 0.015 and *ζ*_1_ (automatic stream) is 0.004. Because, typically the automatic stream will start earlier, sometimes the automatic response will win the competition, while at other occasions the intentional response will win. In addition, the system also allows speed-accuracy control by changing the response threshold; this is based on information about accuracy of performance and a mechanism to change the speed-accuracy trade-off (SAT) settings by means of production rules. The motor parameters of the system indicate whether the system is initiated (NONE, ORAL or MANUAL), its status (BLOCKED which means no access, UNBLOCK available, DONE or no longer available), the current response if any, the threshold, the episode of last threshold change, the SAT-control parameter and its strength. For completeness, it must be added that this kind of response competition is not used in a recall context, but it is in recognition contexts as well as in non-memory tasks.

The general system also allows temporary activation of *‘internal stimulus events’* (ISE). For example, after execution of a response, the system can generate internal feedback based on the response emitted (whether the response is the same as the one supported in the intentional stream). The event has a name or NONE, an indication whether it is in use, a start and an end, the last response registered and evaluation of the response. When such an event is used for feedback, the information contained in the internal stimulus event is passed on to production rules that match the ISE.

### PERFORMING A SIMPLE TASK

**Figure 2:**
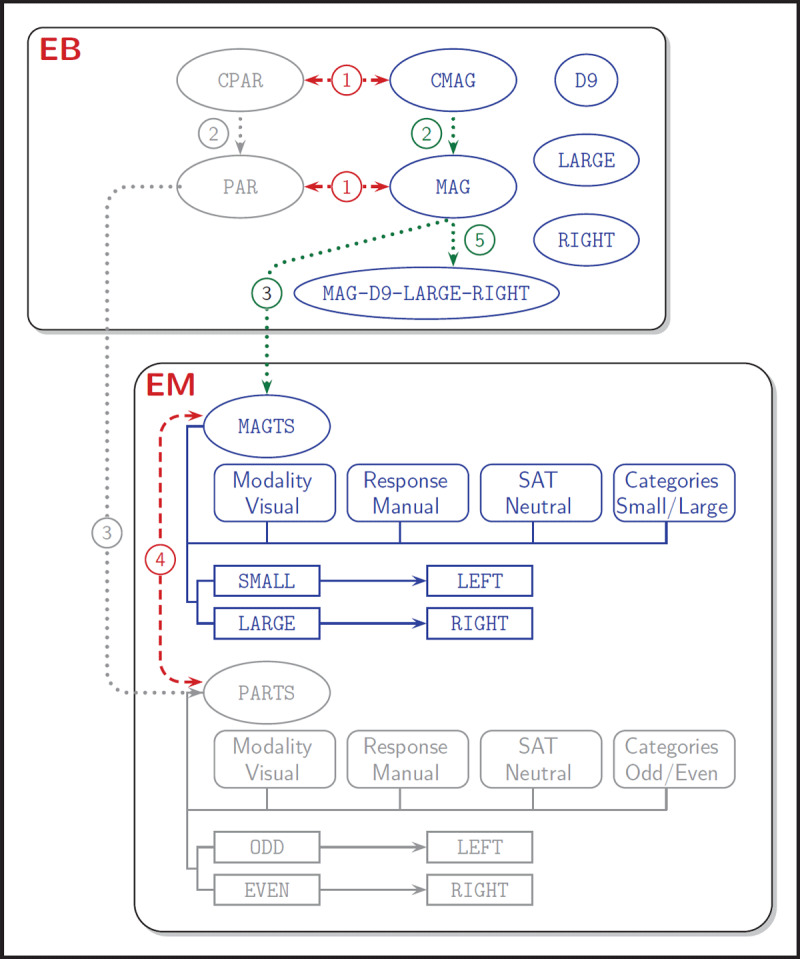
Illustration of events as they occur on a single task-switching trial. The figure displays the events represented in the the Episodic Buffer (EB) and in Executive Memory (EM). Over the trial, the magnitude cue (CMAG) is presented which triggers instantiation of the task goal (MAG). Later on the target (D9) is presented, which allows acivation of the task set (MAGTS) in EM. Next, the target is categorised as LARGE and in combination with the task set this leads to activation of the corresponding response (RIGHT). The goal, target, category and response are bound together (MAG-D9-LARGE-RIGHT). Finally, the response is executed.

In order to clarify how all the parts of the modelling work together, here a description is given of what happens when executing a trial as specified in ***Table 2***. It is assumed that before the start, instructions have been given that digits will be presented for magnitude judgement, such as for example that the applicable mapping for task set MAGTS is SMALL-LEFT. These instructions are available via the function tskinstr and accessible to the production rules. As this example is based on a simulation of the model, the cycle numbers at which events happened are mentioned. ***Figure 2*** illustrates the events described here; the figure contains some numbered arrows to indicate the order in which the events are occurring; green arrows for activation and red ones for inhibition.

At time 0, a black CROSS at position (4,4) becomes available, but the fixation is at a random position in space, and a saccade is required to fixate position (4,4). As no rules match the presence of the cross, nothing happens until cycle 50. At that moment, the magnitude cue is shown (CMAG), and a rule to create an instance matches and is executed (lateral inhibition is applied to a competing cue, arrow 1). Some cycles later (c62)^3^ the instance becomes accessible and is matched by a rule to load the corresponding goal MAG (arrow 2). At the time this goal becomes accessible (c89), no new events occur and no new rules are activated.

At c140, the target object D9 is presented. The presence of the object, matches several rules. One of these is the rule to create an instance of the object in EB. It is instantiated in EB at position (4,5) to become accessible from c157 on. Also the production rule for setting up a task set matches the presence of the object and is activated at c141 (arrow 3) and the activation level of the task set grows over the next cycles (while a competing task set is laterally inhibited; arrow 4). Based on the presence of task set parameters, from c143 on also the motor system is initialised to manual responding, but blocked for now. As D9 becomes accessible at c157, the joint accessibility of the goal and the object result in the creation of a special bind instance (MAG-D9) to become accessible at c165. Meanwhile at c159, the categorisation LARGE is instantiated and will be accessible at c178. Next (c174), task set parameter RSPCTL gets the value UNBLOCK and this also unblocks the motor system. The mapping rule LARGE-RIGHT is added to the task set (c178), and the category is added to the bind (c179). Response RIGHT is instantiated (c180) to become accessible on c206, and at that time a rule detecting this response triggers an automatic response stream to execute this response. Next, the bind is completed (c207) with the response and EB contains now the complete bind MAG-D9-LARGE-RIGHT (arrow 5). This bind matches a rule (c209) to execute the response RIGHT (intentional stream), and the tendency to respond RIGHT now will build up until it reaches the threshold value, which occurs at c230 and is registered at c231.

The response is conveyed to the environment and the environmental states are now adapted, the cue ends at c232, the object stops also at that cycle and now an empty interval of 50 cycles follows until c281. The motor system changes its status to DONE. The system detects that the last response was the one of the intentional stream and generates the internal feedback event CORRECT. The situation now matches the condition for the learn function to become active. This function performs several actions in a series of steps. First, it adapts the strengths of the production rules that were active during this trial (c233). Next (c234) the learn function checks whether the assocation rule D9-RIGHT already exists, if not (c235) it creates a new rule of the association. Subsequently, it inhibits the response (c236), and removes the internal feedback (c237). When this is finished, the components of the bind are inhibited and finally the bind is desintegrated (c240–c244). The system is ready for the next trial. The complete series of events discussed in this example only involve the components dLTM, pLTM, sensory memory, EB and EM.

### PERFORMING A SERIAL RECALL TASK

**Figure 3:**
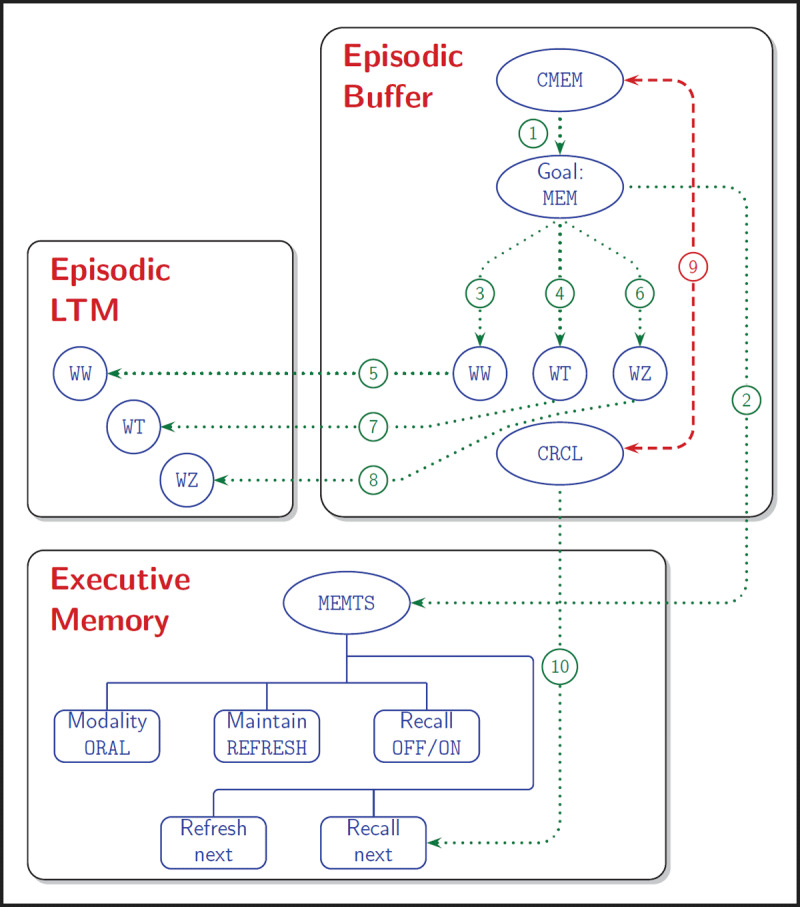
Example of contents of the episodic buffer, the executive memory module and episodic long-term memory during a trial of a serial recall task. After the cue to start acquisition (CMEM) is available in the episodic buffer, the memorisation goal is instantiated (arrow 1), which triggers implementation of the memorisation task set in executive memory (arrow 2). Next, one by one the memoranda are presented and become instantiated in the episodic buffer (arrows 3, 4, 6). After a sufficient amount of refreshing, these memoranda can be represented in episodic LTM (arrows 5, 7 and 8). At the end of the learning phase, a recall cue is presented and activated in the episodic buffer; this laterally inhibits the learn cue (arrow 9), and triggers an action change in the task set including a shift from ‘Recall=OFF’ to ‘Recall=ON’ (arrow 10). After which recall proceeds and finishes.

For this illustration, a small example is used of a single memorisation and recall trial starting with the presentation of a start signal (CMEM) on position (4,3) at time 0, followed by three letters on position (4,4) for recall (WZ at c100, WQ at c200, and WT at c300) and then at c400 on position (4,3) a recall signal (CRCL) for a 500 cycle recall period. Because what happens with the letters is bound to be repetitive, only for the first letter all details of processing will be mentioned. ***Figure 3*** shows how EB, EM and eLTM are used to perform this recall task, keeping in mind that as always also sensory memory, dLTM and pLTM are required. The sequence of events depicted in the figure are indicated by numbered arrows as mentioned in the following description.

First a saccade is programmed to the center of the visual area, so that at c10 the start signal is detected and instantiated as a memorisation cue in EB. The cue becomes accessible at c29 and allows (arrow 1) the memorisation goal (MEM) to be loaded and become accessible at c52. At that time the rule for task set instantiation matches (arrow 2) and the MEMTS task set is loaded with six parameters with default values (namely ORAL for memory modality, NONE for response modality, NORF for maintenance mode, OFF for recall, L0 for chunking size, and ALL for refreshment schedule). On the cycles that follow, rules for setting the task set parameters are triggered so that at the end of the preparation period (until c99), response modality is ORAL, maintenance mode is RF, and the other parameters have not changed. Due to the change of the maintenance mode, the rule for loading the refreshment action matches which results in setting up this action at c86.

Now at c100 the first letter appears (arrow 3), and a saccade is under way. At c106 letter WZ is instantiated in EB with mark TBR (to-be-recalled) and is accessible from c128 on. The rule to start refreshment is triggered at c130 and starts refreshment/consolidation at c132. At this point the activation level of WZ is 0.25 and at c199 it has increased to 0.57. At c200 (arrow 4), WQ is instantiated and becomes accessible at c218; until then refreshment is confined to the first letter only. From c222 refreshment alternates between the two letters and at c299, both have an activation level of 0.64. Meanwhile, the strongest letter is consolidated in eLTM (arrow 5). WT is instantiated at c301 (arrow 6) to become accessible at c323. At c399 all three letters have been regularly refreshed and have respective activation levels of 0.72, 0.76 and 0.42 and they have traces in eLTM with respective strengths of 0.15, 0.08 and 0.02 (arrows 7 and 8).

As a saccade was going on, the recall cue is instantiated at c405 and becomes accessible at c425; it laterally inhibits the CMEM cue (arrow 9). The rule to change the recall parameter to ON matches the presene of the new cue at c426 (arrow 10) and the retrieval action is called for from c432 on; it takes until c442 for the action to be uploaded and become active. The first recall attempt starts at c443. The next item to be recalled is found to be WZ; this is based on a combination of the age (number of cycles since instantiation) and the activation of the instance. At this point WZ is accessible and will be available from c502 on; meanwhile refreshment continues. At c508 WZ is sent out for oral respnding and recall occurs at c509. Meanwhile the next letter WQ is found and is accessible and will be available from c542 on, and is sent to output at c547. The last letter is sent out at c607, which makes recall complete and in the correct order.

This example used only refreshment and hence did not call on the phonological loop to maintain and retrieve the letters. However, within WMDEC it is possible to combine refreshment, rehearsal and revival in maintenance and recall. In (serial) recall, first the next to be recalled item is located in EB. When it is found and revival is on and the fail count is 0, first access in VSM is tried; if access fails, the VSM-fail count is increased and four cycles later a new attempt can be made. If access succeeds, recall proceeds on the basis of VSM. Similarly, if instead rehearsal is on, the same attempts are made on the basis of the phonological loop. On the next attempt for the same item, as long as the number of failures is within the accepted range (less than 3), recall is tried from EB and if that fails eLTM is tried; if either of these succeeds, the item is recalled, otherwise if the failure rate is too high, failure is registered and the next item is tried.

## APPLICATIONS TO MULTI-TASKING

Due to the conceptualisation of WMDEC as a model that not only includes facilities for temporary memory maintenance but also for support of goal-directed actions, the model should be able to predict multi-tasking costs in a variety of situations. Because the model maintains task sets to support goal-directed action, the model should predict the costs incurred due to task set change as well as the benefits of advance preparation. In other words, the model should predict (1) the occurrence of task switch costs both in terms of response time and response accuracy, (2) faster and more accurate responding in trials with a longer cue-target interval duration, and (3) smaller switch cost sizes with a longer cue-target interval duration. In a similar vein, the model should predict not only the costs due to task switching but also costs associated with changes to task parameters such as orientation of attention.

Apart from task switching performance, the model should also account for costs associated with dual-tasking not only in straightforward dual-task situations but also in contexts where the dual-task costs may vary over conditions. Hence, the model should account for the costs of cognitive load on memory performance in a dual-task setting (e.g.,. Barrouillet et al., 2007). But also in more complex operational contexts, the model should account for the observations. For example, it has been observed that the requirement to maintain information in memory does not suffer from the requirement to perform cognitive tasks during memory maintenance. A context in which this occurs, is provided by the task span procedure described by Logan (2004). Finally, the model may also be expected to account for the subtle costs in memory performance due to the increased cognitive load associated with frequent switching between two (or more) tasks during memory maintenance (e.g., Liefooghe, Barrouillet, Vandierendonck & Camos, 2008).

### METHOD

The computational version of the model will be used to simulate performance in all the situations mentioned. For each situation a separate program specifying all the environmental events, the experimental procedure and the interactions with the model is developed. This program sets up a simulation for the required number of statistical subjects and reports the model’s responses for every trial in the procedure. These simulated data are then aggregated and subjected to statistical analyses in order to verify whether the model generates the expected effects. In this process, the model parameters are fixed and are the same for all the applications of the model. However, in order to produce inter-subject variability, the value of six parameters is selected randomly within bounds. It concerns the parameters *α* (activation growth in EB and EM), *β* (lateral inhibition rate in EB and EM), *φ* (initial activation level in EB and EM), *τ* (inhibition rate), *θ* (response threshold) and *ζ* (the drift rate of the intentional response tendency). The bounds of variation of these parameters are displayed in ***Table 1*** in the columns labeled ‘Low’ and ‘High’. The selected parameter value is obtained as follows. Let *m* be the standard value of the parameter and let *l* and *h* be the low and high end of the range of variation, and let *r* be a gaussian random number *r ∈ N*(0, 1), a value *v* is calculated as *v = r(h – l)/6 + m* with the restriction that *v* could not be smaller than *l* and not larger than *h*. This procedure ensures that the variability of the generated data is quite large, so that it is meaningful to perform statistical analyses to test whether the expected effects do occur and are reliable. Comparison of these statistical outcomes to the expected or predicted outcomes constitutes a first part of the model evaluation.

With fixed parameter values, even with the allowed variability, it cannot be expected that the model will yield a numerically close fit to the data. However, the means obtained in the simulation and the means in the observed data should show the same pattern. This is tested by means of the product-moment correlation which is tested for statistical significance.^4^

### STUDY 1. TASK SWITCH COST AND PREPARATION EFFECTS

The predictions regarding switch cost and its reduction due to preparation (for more details about these predictions and their motivation, see top panel of ***Table 3***) were verified by simulating the conditions of Experiment 2 as reported by Logan and Bundesen (2003). In this experiment, twenty subjects were presented with three tasks to be applied to digit and digit word stimuli. The three tasks were magnitude judgment (smaller or larger than 5), parity judgment (odd or even), and form judgment (digit or digit word). The stimuli were the digits 1–9, excluding 5 and the digit words one to nine, excluding five. Each trial started with a cue that was followed by a target (digit or digit word) with cue-target intervals (CTI) ranging from 0 to 900 ms in steps of 100 ms. The cues, tasks and stimuli were produced by a design that included 3 (cues) × 16 (targets) × 10 (cue-target intervals) for a total of 480 trials. This block was repeated once with a mask and once without a mask. The order of the trials was randomised per subject. The simulation did not include the block with masked stimuli.

**Table 3:** WMDEC predictions regarding task switching performance as tested in Studies 1 and 2 and a motivation why the model supports these predictions.


PREDICTION	WHAT^a^	WMDEC EXPLANATION

Study 1: Task preparation and switch cost (SC)		

Task preparation	*P_+_ > P_–_*	Cue processing and goal activation may complete before the task stimulus (target) is presented

Task switch cost	*P*_tr_ > *P*_ts_	On switch trials but not on repetition trials, a new task set must be activated and configured in EM

SC reduction	*C_t+_ > C_t–_*	Preparation effect is smaller on repetition trials because less preparation is required

Residual SC	*C_t_ >* 0 (always)	Task set activation and configuration can only complete after the target is presented

Study 2: Task and dimension switching		

Dimension SC	*P*_dr_ > *P*_ds_	When the task repeats, a dimension switch requires changes to the active task set

Task/dimension SC	*C_t_ > C_d_*	A task switch requires a completely new task set including a dimension configuration, whereas a dimension switch requires only a change to a task set parameter when the task remains the same

Alternative	*C_t_ = C_d_ = C_t+d_*	If it is assumed that every combination of task and dimension requires its own task set, any change (task, dimension or both) requires activation of of a new task set


*^a^* Predictions are formulated in terms of performance (*P*) as a function of preparation time (*+* for longer, – for shorter) or transition (ts for task switch, tr for task repetition), where higer performance corresponds to faster RT and fewer errors. Some predictions are formulated as a performance difference score, namely switch cost (*C_t_ = P*_ts_ – *P*_tr_ for task switch cost, *C_d_* = *P*_ds_ – *P_d_*_r_ for dimension switch cost).

For the simulation of this experiment, dLTM contains the digits 1–9 (D1–D9) and the words one to nine (W1–W9), the cues for the magnitude, parity and form tasks (CMAG, CPAR, CFRM), the three corresponding goals (MAG, PAR, FRM) and task sets (MAGTS, PARTS, FRMTS) with their respective parameter sets (MAGPAR, PARPAR, FRMPAR) and mappings (MAGMAP, PARMAP, FRMMAP), the categories associated with these tasks, namely SMALL, LARGE, ODD, EVEN, DIG, WRD, and the responses (LEFT, RIGHT). Likewise, pLTM must contain the rules to set up the category-response mappings, categorisation, response generation, and binding. What happens during the trials corresponds with the description given in the section ‘Performing a simple task’.

Because preparation starting from the cue and leading up to the activation of the task set takes some time, it is evident that when the target is presented before the preparation is complete, responding will take longer than when target processing starts after completion of the preparation. Likewise when the same task is repeated, cue processing, goal uploading and task set activation will not take long as these representations are still present with some strength in the episodic buffer.

#### Results and Discussion

The model was applied to this design and replicated on 30 statistical subjects. ***Table 4*** displays the simulated RTs as a function of repetition vs. switch and CTI. The simulation revealed faster responses on repeat trials (M = 829 ms) than on switch trials (M = 891 ms), *F*(1,29 = 94.00, *p* < 0.001, *η_p_*^2^ = 0.76. Responses were faster with longer cue-target intervals (varying from 1030 ms at CTI 0, to 826 ms at CTI 900), *F*(9,21) = 203.05, *p* < 0.001, *η_p_*^2^ = 0.99. These two factors did interact; this is consistent with the switch cost being smaller at longer intervals, *F*(9,21) = 28.06, *p* < 0.001, *η_p_*^2^ = 0.92. ***Table 4*** shows this decrease in switch cost and shows that the switch cost did not disappear at the longer CTI intervals; in other words, a residual switch cost remained present, which is consistent with findings reported in the literature.

**Table 4:** Means of observed and predicted RT (in ms) as a function of task transition (repeat vs. switch) and cue-target interval (CTI) in Experiment 2 of Logan & Bundesen (2003).


	0	100	200	300	400	500	600	700	800	900

Observed RTs										

Repeat	1132	976	929	850	820	790	760	764	769	750

Switch	1463	1330	1237	1127	1063	1013	995	964	918	917

Predicted RTs										

Repeat	940	858	819	819	807	817	808	808	809	802

Switch	1121	996	906	854	839	841	841	836	844	831


These results were largely mimicked in the analysis of the accuracy data, which showed an overall switch cost (M = 99.5 percent correct for repetitions and M = 98.1 percent for switches), *F*(1,29) = 22.64, *p* < 0.001, *η_p_*^2^ = 0.44, a main effect of CTI (M varied from 95.2% correct at CTI 0 to 99.5% percent at CTI 900), *F*(9,21) = 2.65, *p* < 0.05, *η_p_*^2^ = 0.59, and an interaction of transition by CTI, *F*(9,21) = 3.00, *p* < 0.05, *η_p_*^2^ = 0.56.

As can be seen in ***Table 4***, the RTs predicted by the model decrease with CTI but the slopes of these decreases were smaller in the predicted than in the observed RTs. The fit of the predictions to the data was assessed by means of the correlation between the predicted and observed means. For this application, the correlation amounted to 0.90, *t*(18) = 8.79, *p* < 0.001 for RT, and 0.52, *t*(18) = 2.60, *p* < 0.05 for accuracy.

This first application of the WMDEC model shows that the model correctly generates switch costs, that performance improves with a longer preparation interval and that the switch cost is reduced with longer preparation as well in the RT as in the accuracy measure. Moreover, the switch cost does not disappear at long preparation intervals. It is important to note that, numerically, the switch costs and the preparation effects were larger in the data than in the simulation. These differences may signal that the model is missing some important aspects of what is going on in task switching, but it is also possible that some of the assumptions made to run the simulation entail an unwarranted simplfication. In fact, in this experiment subjects applied three tasks to two subsets of qualitatively different stimuli. It was assumed that the three tasks, magnitude, parity and form judgment were equally difficult; it was also assumed that these three tasks were equally difficult when the stimuli were digits and when the stimuli were digit words; and it was also assumed that there were no differences in task preparation in all these conditions. The reason for these assumptions was that no separate data per task and per stimulus type were available. It is well known that there are performance differences between these tasks and stimuli, so that with more variation in task difficulty, also more variation in task switching and task preparation effects could be present. Hence, before accepting the conclusion that the model should be improved, it would be helpful to perform the simulation on a more detailed data set.

### STUDY 2. TASK AND DIMENSION SWITCHING

In situations where tasks can be applied to two different aspects of a stimulus, attention must be oriented to the currently relevant aspect. In a task switching context this means that over trials as well the task as the relevant stimulus component can change. This situation typically occurs with Stroop stimuli such as colour words printed in colour, where attention can be to the word or to the colour of the print (Stroop, 1935). In a task switching context, typically as well the task as the relevant stimulus aspect are cued. When the task changes, a new task set must be activated and a switch cost will occur, but this implies that the orientation of attention parameter must be set as required for the task, so that a task change always involves a resetting of both task and orientation of attention. In contrast, however, when the task remains the same, but the orientation of attention must be adapted, this task set parameter requires a change, which should result in a smaller cost for attention-only changes than for task changes with or without a change in the dimension of attention. Such an analysis predicts that there will be both task switch costs and dimension switch costs, and that the task switch cost will be larger than the dimension switch cost (see bottom panel of ***Table 3***).

These expectations are at variance with the findings reported by Allport, Styles and Hsieh (1994), who found that in a list procedure, alternating lists with any change (only the task, only the dimension or both task and dimension) always required more time than no-change lists, while no differences were observed between these three types of change. However, also other patterns of findings have been reported (e.g., Hübner, Futterer & Steinhauser, 2001; Kleinsorge & Heuer, 1999; Kleinsorge, Heuer & Schmidtke, 2001, 2002, 2004); some of these findings are more or less in line with the expectations formulated above. In order to clarify these contradictory findings, which are possibly due to variations in designs, procedures and materials, Vandierendonck, Christiaens and Liefooghe (2008), studied the task and dimension switch costs by systematically varying materials and cue-target intervals within one single procedure, namely the task-cuing procedure. The four experiments in the latter study replicated the findings originally reported by Allport et al., confirming that irrespective of the variations in materials, and procedure, any change in the task or the dimension or both, resulted in a switch cost and the size of the cost did not differ among the three kinds of changes. Clearly, on the basis of these findings, it would be predicted that task and dimension switch costs are equally large and it would not make any difference whether only one (either the task or the dimension) or two (both task and dimension) change. These predicitions, which follow from a different strategy to cope with combined task and dimension changes, are also presented in the bottom panel of ***Table 3***.

The computational version of WMDEC was applied to the first two experiments of the latter study, in which the cue-target interval was varied, while the materials were fixed. In these experiments, the stimuli consisted of the digits 2, 3, 5 and 6 shown as 2, 3, 5 or 6 digits in a playing card-like configuration. For example, when the digit 2 is shown at the four corners of a square (i.e., four times), with attention oriented towards the individual digits, the value observed is two, while when attention is oriented towards the collection of digits shown, the observed value is four. The tasks in these experiments involved either a categorisation on the basis of magnitude (smaller or larger than 4) or parity (odd or even), and the task could be applied to the digits or to the number of digits shown. This was implemented by simultaneously presenting a digit (D2, D3, D5, D6) left of center and a word (W2, W3, W5, W6) right of center.^5^ Four different cues were used, namely CMAG, CMAGN, CPAR, and CPARN, where the cues ending on ‘N’ referred to the number of element (words) and the other cues referred to the digits. Two goals and corresponding task sets were used, namely MAG and PAR.

#### Experiment 1: Cue and target simultaneous

The first experiment of the study of Vandierendonck et al. (2008) tested task and dimension switching with a cue-target interval of 0 ms, which limits the design to a 2 (Task transition: repeat or switch) × 2 (Dimension transition: repeat or switch) repeated measures design, with a composite cue indicating which task and which dimension is required. The leftmost columns of ***Table 5*** show the observed and the predicted means, under the header “CTI 0”.

**Table 5:** Means as a function of Task transition (Repetition/Switch) and Dimension transition (Repetition/Switch). Observed data in Experiments 1 and 2 of Vandierendonck et al. (2008) and the predicted means under two different sets of assumptions within the context of the WMDEC model.


Task	CTI 0		CTI 300		CTI 1000	
		
	REP	SWITCH	REP	SWITCH	REP	SWITCH

Observed means						

Rep	1752	2020	1485	1739	1073	1326

Switch	2042	2005	1728	1673	1320	1294

Prediction 1						

Rep	1126	1174	974	978	982	976

Switch	1253	1255	1005	1008	989	975

Prediction 2						

Rep	1097	1314	974	1081	975	1040

Switch	1309	1300	1081	1068	1023	1023


##### Results

As expected, the pattern of predicted RTs did not match the observed pattern. In order to distinguish between the different views expressed in the literature, it is useful to decompose the effects of task and dimension switching into the following orthogonal contrasts:

1. complete repetition versus any change (the ‘flat’ structure contrast, cf. Allport et al., 1994);
2. within the changes: a change in one component (task or dimension) versus a change of both
3. within the changes of a single component: a task versus a dimension change.

In the findings of Vandierendonck et al. (2008), the first contrast was the only significant one, and explained 99% of the variance among the four means. In the model’s predictions, all three contrasts were significant, respectively *F*(1,19) = 188.60, *p* < 0.001, *η_p_*^2^ = 0.91, *F*(1,19) = 153.84, *p* < 0.001, *η_p_*^2^ = 0.89, and *F*(1,19) = 171.52, *p* < 0.001, *η_p_*^2^ = 0.90; they explained respectively 64, 9, and 26% of the variance. The correlation between observed and predicted mean RTs amounted to 0.73, *t*(2) = 1.53, *p* = 0.27.

Clearly, the model’s predictions are more in line with the results reported, for example, by Kleinsorge et al. (2002), and indeed the assumptions made by these authors strongly resemble the assumptions within the WMDEC model.

Given that production rules are based on learning and experience, for a participant in such an experimental setting, it may be more advantageous to treat the four combinations of task and dimension as separate goals, namely ‘digit magnitude’ (MAG), ‘number magnitude’ (MAGN), ‘digit parity’ (PAR) and ‘number parity’ (PARN). In order to attain these goals, corresponding task sets are required. A second test of the model was therefore performed based on this different way to conceptualise the set of goals. The results are shown in ***Table 5*** in the panel ‘Prediction 2’. The pattern of these results is much closer to the observed pattern. The first two contrasts attained significance, *F*(1,19) = 1133, *p* < 0.001, *η_p_*^2^ = 0.98, and *F*(1,19) = 15.49, *p* < 0.001, *η_p_*^2^ = 0.44, respectively. The third contrast was not significant, *F*(1,19) = 1.54, *p* = 0.23, *η_p_*^2^ = 0.08. The three contrasts accounted for respectively 99.7, 0, and 0% of variance among the means. Correlation of observed and predicted mean RTs was 0.998, *t*(2) = 22.33, *p* < 0.01.

#### Experiment 2: Variations in preparation time

The second experiment in Vandierendonck et al. (2008) compared task and dimension switching with CTIs of 300 ms and 1000 ms. These longer CTIs allow for time to prepare the upcoming task before the target is presented. As expected, a reduction in switch cost was observed, but the pattern of findings was in both conditions the same as in the first experiment, namely a confirmation of the ‘flat’ structure.

##### Results

The same variations in the model’s implementation as used for the first experiment were tested for this experiment. The observations and predictions are displayed in the columns ‘CTI 300’ and ‘CTI 1000’ of ***Table 5***. In line with expectations and the results obtained for Experiment 1, the simulations based on the first set of assumptions did not provide a close match to the data. The simulation results revealed significant main effects for all three factors. Importantly, the effects of CTI, Task transition and their interaction were reliable, respectively *F*(1,18) = 6.37, *p* < 0.05, *η_p_*^2^ = 0.26, *F*(1,18) = 17.44, *p* < 0.001, *η_p_*^2^ = 0.49, and *F*(1,18) = 18.02, *p* < 0.001, *η_p_*^2^ = 0.50. This confirms that the model also predicts the preparation effect and the reduction of switch cost with preparation duration. Overall, the first two contrasts did not attain significance, *F*(1,18) = 3.10, *p* = 0.095, *η_p_*^2^ = 0.15, and *F <* 1, but the third contrast did *F*(1,18) = 5.77, *p* < 0.05, *η_p_*^2^ = 0.24. These contrasts explained respectively 26, 4, and 70% of the variance among the means. The correlation between the observed and predicted mean RTs amounted to 0.51, *t*(2) = 0.84, *p* = 0.49.

As expected, the implementation based on the usage of four different task sets for the four task-dimension combinations, revealed the preparation and task switch reduction effects: *F*(1,18) = 165.41, *p* < 0.001, *η_p_*^2^ = 0.90 for the factor CTI, *F*(1,18 = 29.07, *p* < 0.001, *η_p_*^2^ = 0.62 for the task switch cost, and *F*(1,18) = 55.99, *p* < 0.001, *η_p_*^2^ = 0.77 for their interaction. Only the first of the three orthogonal contrasts attained significance, *F*(1,18) = 36.38, *p* < 0.001, *η_p_*^2^ = 0.67, and respectively *F*(1,18) = 3.76, *p* = 0.07, *η_p_*^2^ = 0.17, and *F <* 1 for the other two contrasts. They accounted for respectively, 98, 2 and 0% of the variance among the means. The correlation between observed and predicted mean RTs amounted to 0.78, *t*(2) = 1.77, *p* = 0.22.

#### Discussion

The two variants of the model tested in this application of WMDEC to task and dimension switching confirmed the results already observed in the model’s application to the data of Logan and Bundesen (2003):

1. the model predicts the switch cost: responses on task switch trials were slower than on task repeat trials in all applications and variants thus far;
2. the model predicts faster performance when there are opportunities to prepare for the upcoming task before the target stimulus is presented; this performance gain occurs for both switch and repeat trials;
3. the model predicts a reduction of the switch cost with longer advance preparation intervals. Although both switch and repeat trials benefit from the advance preparation, the gain is larger for the switch conditions with as a result a larger decrease of the switch cost with longer preparation intervals.
4. The model predicts a residual switch cost as the cost does not disappear after a long preparation interval.

The experiments about task and dimension switching reported in the literature have revealed different patterns of results. Assuming a hierarchical task set organisation with the task goal situated at the top and the dimensional representations at a lower level, it is predicted that the dimension switch cost is smaller than the task switch cost. This particular prediction was made also by the model based on the assumptions that dimension is subordinate to task. In contrast, when it was assumed that dimension and task are represented at the same level as in the second application of the model, the flat structure observed in a number of reports was confirmed. These two simulation outcomes of the same data on the basis of the same model raise some issues for discussion. These are deferred to the General Discussion.

Apart from this issue, it should also be noted that the preparation effect present in the simulation of the second experiment is rather limited. Whereas the data show an RT drop of about 300 ms from CTI 0 (Experiment 1) to CTI 300 and another drop of at least 300 ms from CTI 300 to CTI 1000, this is not mimicked in the simulation which shows only a big drop from CTI 0 to CTI 300 and a very small drop to CTI 1000. It may be suspected that this difference between simulation and data is a consequence of the implementation of the number stimuli in the simulation as number words so that the processing time of these stimuli is likely to be shorter than in the data.

## STUDY 3. SIMPLE DUAL-TASKING

In contrast to task switching which concerns flexible transitions from one task to another, dual-tasking requires the simultaneous activation and execution of two or more tasks. In principle, dual-tasking can involve any two tasks, but in the context of working memory research typically a memory maintenance-and-recall task is combined with another task. As WMDEC is a working memory model, it should be able to perform such coordination with the same costs as observed in typical experimental settings. In the present report, only one such experiment is considered because in a later section, more complex forms of dual-tasking will be addressed.

A study by Barrouillet et al. (2007) reports several experiments designed to test their Time-Based Resource Sharing (TBRS) model. This model assumes decay of working memory contents unless these contents are reactivated by means of attentional refreshing. The model assumes that central attention can be assigned to only one task at a time, so that dual-tasking requires rapid switching between the tasks that call on this resource. If during the maintenance intervals, other tasks have to be performed, these tasks occupy central attention for as long as needed to complete the task. As a consequence attention is not available for refreshing. The larger the proportion of the maintenance interval is occupied by the secondary task (i.e., the higher the cognitive load), the less opportunities are present for refreshing and the poorer recall will be. So, when the secondary task is easy (occupies attention for a shorter period) recall will be better than when the secondary is more difficult (occupies attention for a longer period).

In their Experiment 3, which is selected for the present simulation test, these authors used a complex span procedure. After the presentation of every to-be-remembered element (letters), a short period for refreshing was available, but in this interval a digit task intervened which required to make a parity judgment (odd or even) or a location judgment (is above or below the center of the screen). They presented series of letters with lengths varying from 1 to 7 where each letter was presented for 1500 ms and after a delay of 500 ms either 4, 6 or 8 digits were presented during an interval of 6400 ms, such that in the 4-digit condition, each digit was shown for 1067 ms followed by a 533 ms empty period; in the 6-digit condition, presentation duration was 711 ms on 356 ms off, and in the 8-digit condition, 533 ms on, 267 ms off. They found that memory span decreased with the number of intervening digit decisions and that the spans were shorter with the more difficult of the two tasks. A WMDEC simulation should reproduce these results, corresponding to the predictions that memory span is lower when the secondary task requires more time to execute, as when the task is more difficult, or takes a larger proportion of the available time or must be executed more frequently. These predictions are listed in ***Table 6***.

**Table 6:** Overview of WMDEC predictions in a dual-task situation with execution of tasks varying in difficulty and frequency during the maintenance period in a complex span setting.


PREDICTION	WHAT^a^	WMDEC EXPLANATION

Study 3: Secondary task in maintenance interval		

Task difficulty	*M*_dif_ < *M*_easy_	A more difficult task takes more time to complete than an easy task; as a consequence refreshment of to-be-remembered elements is blocked for a longer time

Number of tasks	*M*_high_ < *M*_low_	When more tasks have to be completed during the maintenance interval, less time is available for refreshment of to-be-remembered elements


*^a^* In dual-task context, a secondary task is present during maintenance and/or retention intervals of a serial recall task, which is scored as the number of correctly recalled elements in the correct serial position (i.e., memory span, *M*). This measure is observed in a difficult (*M*_dif_) or in an easy (*M*_easy_) condition, with a high or a low number of intervening tasks (*M*_high_ and *M*_low_).

Like the experiment, the simulation used letters (WA–WZ) for the recall task and digits (D1–D4,D6–D9) for the parity and the location tasks; these digits were positioned either above or below the centre of the 9 by 9 visuospatial matrix. The recall task was announced by presentation of a cue (CMEM) at the start of the trial, followed by a letter, and the 4, 6 or 8 digits, after which a signal for recall was shown (CRCL). The timing was the same as in the experiment; that means, the times used in the experiment were divided by 10 to obtain the cycles at which the planned events were to occur. In order to achieve coordination of the memory task on the one hand and the judgment task on the other hand, an instance DUAL of type COORD was kept active during all the dual-task trials (not during the single-task practice trials, which were also included in the simulation.)

### Results and Discussion

The 2 (Tasks: location vs. parity) × 3 (Number of tasks: 4, 6 or 8) between-subjects design was implemented with 16 statistical subjects per cell. Observed and simulated results are displayed in ***Table 7***. With memory span as dependent variable, the simulation revealed main effects of both factors. When the task was more difficult (parity), span was shorter (M = 4.23) than when it was easier (location; M = 4.65), *F*(1,90) = 6.76, *p* < 0.05, *η_p_*^2^ = 0.07. The linear trend of memory span over the number of tasks within the maintenance interval was significant, *F*(1,90) = 4.97, *p* < 0.05, *η_p_*^2^ = 0.05. That these effects were indeed due to the durations of the secondary task can be seen in the observation that the RTs of the digit categorisation task were longer for the parity task (M = 686 ms) than for the location task (M = 612 ms), *F*(1,90) = 43.52, *p* < 0.001, *η_p_*^2^ = 0.33. RTs did however not reliably decrease with the number of tasks in the maintenance interval, *F <* 1, but more importantly, the total time during which the maintenance interval was occupied by the task increased with the number of tasks, 2,567 ms, 4,017 ms and 5,092 ms for respectively, series of 4, 6 or 8 tasks, *F*(2,90) = 1725.22, *p* < 0.001, *η_p_*^2^ = 0.98. Whereas the latter increase over number of tasks is important as it drives the decreasing memory span, the observation that RT per task remained stable over number of tasks in the simulation in contrast to an RT decrease in the data, suggests that subjects in the experiment were more flexible by speeding up their responses in order to gain more time for refreshment.

**Table 7:** Observed and simulated means (standard deviations between brackets) for the secondary task RTs, total processing time, and memory span in the complex span task design with memory list lengths from 1 to 7 and with tasks varying in difficulty (parity vs. location judgment) executed during the maintenance and retention periods.


NUMBER OF STIMULI	PARITY			LOCATION		
	
	RT	TOTAL TIME	SPAN	RT	TOTAL TIME	SPAN

Observed						

4	628 (117)	2,467 (400)	5.16 (0.78)	484 (61)	1,928 (233)	5.56 (0.75)

6	551 (53)	3,251 (316)	4.58 (1.23)	387 (41)	2,297 (239)	5.52 (0.62)

8	483 (32)	3,724 (218)	3.69 (0.63)	361 (39)	2,827 (266)	4.60 (0.82)

Simulated						

4	677 (46)	2,707 (185)	4.38 (0.69)	607 (23)	2,427 (93)	4.94 (0.55)

6	715 (49)	4,290 (294)	4.19 (0.81)	624 (45)	3,744 (270)	4.56 (0.61)

8	667 (27)	5,334 (215)	4.13 (0.60)	606 (31)	4,850 (247)	4.44 (0.61)


The correlation between the observed and simulated mean spans amounted to 0.81, *t*(4) = 2.81, *p* < 0.05. For the mean RTs, the correlation was 0.71, *t*(4) = 2.03, *p* = 0.11, and for the total processing time during the maintenance interval the correlation was 0.86, *t*(4) = 3.37, *p* < .05. Even though the correlations were rather high, it must be noticed that the RTs for the location task were larger in the simulation than in the observed data. These results show that WMDEC does indeed predict the costs associated with dual-tasking in the context of a working memory serial recall task. These findings also show that the model, in fact, makes the same predictions as the TBRS model. This is because the crucial mediating condition is the degree of cognitive load. The TBRS model predicts that the higher the cognitive load, the fewer the opportunities for refreshing and the poorer recall will be. While WMDEC is based on different mechanisms and assumptions, the crucial factor consists of the time available to refresh memory items during maintenance. That does not imply that both models will always predict the same effects.

## STUDY 4. LOGAN’S TASK SPAN PROCEDURE

In a seminal paper, Logan (2004) developed a procedure to estimate the number of tasks that can be held in memory and correctly executed in the order of presentation. In analogy to the memory span which yields an estimate of the number of items (letters, words, shapes, etc.) that memory can retain in the correct order, the task span yields an estimate of the number of tasks that can be remembered and correctly executed in the correct order. If task execution and memorisation of the task names call on the same working memory resources, the task span should be substantially smaller than the memory span for the same set of task names.

In a series of experiments, Logan showed that the differences between the memory span and the task span were minimal to nonexistent over a range of variations such as the number of tasks to be remembered/performed, the frequency of switches, and the opportunities for chunking. In Experiment 2 of this series, subjects were requested to remember task names in ordered series of varying lengths, either in a memory setting yielding an estimate of the memory span, or in a memory-and-performance setting in which a series of targets were presented with the requirement for each target to recall the next task in the sequence and to apply the task to the target. In this experiment, the tasks magnitude (‘hi-low’), parity (‘odd-even’) and form (‘digit-word’) had to be applied to digits or digit words from 1 (one) to 9 (nine), excluding 5 (five). Sixty-four subjects performed 48 study-test sequences, in a 2 (Response-stimulus intervals or RSI: 100 or 1000 ms) × 2 (List type: 2468 or 2369) × 2 (Task order: memory span before task span or vice versa) between-subjects design. Span type and List length were within-subjects variables; the list length was varied over four lengths depending on the list type: either lengths of 2, 4, 6, and 8 which was considered to be compatible with chunks of 2 or 4, or lengths of 2, 3, 6, and 9 which were thought to be more compatible with chunks of 3. The task names were randomised with the restriction that all three tasks had to be selected before a task could be repeated.

The findings of this experiment can be summarised as follows (see also ***Tables 9*** and ***10***). The proportion of correctly recalled (memory span) or correctly recalled and performed (task span) lists were very similar at each of the list lengths irrespective of list type and response-stimulus interval. In these proportions, within each list type condition, there were only main effects of list length. The spans were estimated as the point corresponding to a proportion of 0.5 correct. Average memory span was 6.9 compared to 6.4 for the task span. These results are taken to support the conclusion that memory is not traded for task execution.

WMDEC was applied to this experimental design with the same constraints. The simulation used digits (D1–D4, D6–D9), number words (W1–W4, W6–W9) and task names (CMAG, CPAR, CFRM). The same design with the same time restrictions (converted to cycles) were used as in the experiment. It is important to note that in the memory-span block of the experimental session only the memory task is relevant, so that this corresponds to a single-task condition. In the task-span block, the first phase consists simply of storing all the task names in working memory and also involves only one single task. During recall, however, the subject is required to alternate between recall and task execution. More specifically, the subject alternates between, on the one hand, the memorisation task in order to recall the next task name, and on the other hand, one of the three categorisation tasks. Hence, in the recall phase with task execution, not only switching between memory and another task, but also switching between the three categorisation tasks is involved. WMDEC’s predictions for this application are listed in the top panel of ***Table 8***. Basically, WMDEC predicts no important differences in the memory and task span, larger memory and task spans for longer lists up to WM capacity limits, and larger memory and task spans with increasing size of chunking.

**Table 8:** Overview of WMDEC predictions regarding memory and task span in Study 4, and the impact of task switch frequency during maintenance and retention interval on serial recall performance in Study 5.


PREDICTION	WHAT^a^	WMDEC EXPLANATION

Memory and task span	*M(n)* ≃ *T(n)*	As recall of task names calls on EB, whereas task execution on EM, no interference is expected between recall and task execution

Memory span increases with length	*M(L) > M(l), L > l*	For list lengths within capacity, more items are recalled the longer the lists

Task span increases with length	*T(L) > T(l), L > l*	For list lengths within capacity, more of the named tasks will be executed correctly

Memory span and chunk size	*M_C_(n) > M_c_(n), C > c*	The larger the chunks, the more elements can be correctly recalled

Task span and chunk size	*T_C_(n) > T_c_(n), C > c*	When more task names are correctly recalled because of chunk size, more of the tasks will be correctly executed

Study 5: Memory span as function of switch frequency		

Alternations and repetitions	*M*_alt_ < *M*_rep_	Because alternations last longer than repetitions, they block refreshment for a longer time

Few and more switches	*M*_many_ < *M*_few_	As task switches take longer than repetitions, the more switches occur the longer refreshment is blocked; this is the case for tasks presented during maintenance as well as during the retention interval


*^a^* Memory span varies with the length of the to-be-remembered sequence (*M(n)*), where *n* is the number of elements, and also chunking affects the memory span (*M_c_(n)*), where *c* is the size of the chunks. In Study 4, also the task span is measured (e.g., *T(n)*). In Study 4, the memory span is registered under task alternation (*M*_alt_) or task repetition (*M*_rep_), and conditions with many (*M*_many_) or few (*M*_few_) switches.

Because chunking can be used to improve recall, three variations of the WMDEC model were applied in this simulation: one without chunking, one with chunking limited to chunks of two elements, and one with chunking limited to three elements. It is possible to implement a model version that adaptively selects the best strategy for the trial at hand, but it was thought that implementing the three strategies as separate models would provide a better insight in how the model achieves its results. First, the results of these three simulations will be presented; next, a weighted combination of the three simulations will be considered. The results of the three simulations are displayed in ***Table 9***.

**Table 9:** Average proportion correct recall in position as a function of Span type, List type, List length and Chunking in the WMDEC simulations applied to Experiment 2 of Logan (2004).


	LIST TYPE 2468				LIST TYPE 2369			
	
	2	4	6	8	2	3	6	9

Observed								

Memory	0.93	0.84	0.57	0.38	0.95	0.89	0.61	0.36

Task	0.90	0.73	0.51	0.26	0.92	0.84	0.57	0.26

No chunking								

Memory	1.00	0.83	0.13	0.01	0.99	0.99	0.16	0.00

Task	0.98	0.84	0.23	0.08	0.98	0.97	0.26	0.05

Chunks size 2								

Memory	0.97	0.97	0.77	0.50	0.98	0.95	0.79	0.29

Task	0.99	0.97	0.81	0.42	0.99	0.88	0.80	0.14

Chunks size 3								

Memory	0.98	0.95	0.79	0.61	1.00	0.95	0.81	0.49

Task	0.99	0.84	0.89	0.46	0.99	0.99	0.88	0.62


### No chunking: Results and Discussion

In the simulation without chunking of task names, proportion of completely correct performance varied with the length of the memory sequence (see ***Table 9***), for both measures, memory span and task span. In the conditions with the 2468 list type, mean proportion completely correct performance varied with Span type, *F*(1,28) = 15.70, *p* < 0.001, *η_p_*^2^ = 0.36, and decreased over the list lengths, *F*(3,26) = 4716, *p* < 0.001, *η_p_*^2^ = 0.998; the trend was strongly linear, *F*(1,28) = 10487, *p* < 0.001, *η_p_*^2^ = 0.997, accounting for 92% of the variance. Only the interaction of these two effects attained significance, *F*(3,26) = 13.37, *p* < 0.001, *η_p_*^2^ = 0.61. The linear trend over the list lengths also interacted with Span type, *F*(1,28) = 34.31, *p* < 0.001, *η_p_*^2^ = 0.55.

A similar result was obtained in the conditions with the 2369 list type. Mean proportion completely correct performance varied with span measure, list length and their interaction, respectively, *F*(1,28) = 12.60, *p* < 0.001, *η_p_*^2^ = 0.31, with proportions of 0.54 (memory) and 0.56 (task span), *F*(3,26) = 7990, *p* < 0.001, *η_p_*^2^ = 0.999, and *F*(3,16) = 13.37, *p* < 0.001, *η_p_*^2^ = 0.61. List length followed a linear trend, *F*(1,28) = 22861, *p* < 0.001, *η_p_*^2^ = 0.999 (87% of the variance), and it interacted with Span type *F*(1,28) = 36.77, *p* < 0.001, *η_p_*^2^ = 0.57. For both list types, it seems that the slope over list length was less steep for the task span than for the memory span, as can be seen in the second panel of ***Table 9***.

The correlation between the observed and model proportions amounted to 0.93, *t*(30) = 13.58, *p* < .001. A span can be estimated from the average proportions per length by interpolation. In the first list type, the estimated memory span and task span were respectively 4.93 and 5.12. In the second list type, the corresponding values were 4.76 and 4.99. These spans are substantially smaller than those reported by Logan (2004), but the low values are in line with what can be expected when no chunking is present.

### Two-item chunks: Results and Discussion

In a second simulation, an attempt is made to form a chunk when two memory items are available. It should be noted, though, that occasionally but not frequently, the formation of a chunk would fail or come too late. With lists of type 2468, average proportions of correct performance decreased with list length, *F*(3,26) = 378.13, *p* < 0.001, *η_p_*^2^ = 0.98, again with a strong linear trend, but with higher values for the longer lists than in the first simulation, *F*(1,28) = 1100, *p* < 0.001, *η_p_*^2^ = 0.98 (86% of the variance). Memory span and task span did not differ significantly, *F*(1,28) = 0.19, *η_p_*^2^ = 0.01. However, type of span and list length interacted, *F*(3,26) = 4.39, *p* < 0.05, *η_p_*^2^ = 0.34, which was mainly due to a steeper decrease in the task span than in the memory span scores, *F*(1,28) = 5.16, *p* < 0.05, *η_p_*^2^ = 0.16.

Similar effects were observed in the 2369 list type, with a main effect of Length, *F*(3,26) = 1303, *p* < 0.001, *η_p_*^2^ = 0.99, that was linear *F*(2,28) = 1795, *p* < 0.001, *η_p_*^2^ = 0.99 (83% of the variance). Task span proportions were smaller (M = 0.70) than memory span proportions (M = 0.75), *F*(1,28) = 17.94, *p* < 0.001, *η_p_*^2^ = 0.39. Length interacted with span type, *F*(3,26) = 19.37, *p* < 0.001, *η_p_*^2^ = 0.69 as did the linear trend *F*(1,28) = 18.82, *p* < 0.001, *η_p_*^2^ = 0.40 for the same reason as in the other list type. Note that the linear trend over list length in this simulation has a steeper decrease for the task span than for the memory span, while this was the opposite in the simulation without chunking. It would seem that the gain from using chunks is larger for the memory span than for the task span.

Correlation between observed proportions and the model proportions based on chunks of size two amounted to 0.93, *t*(30) = 13.34, *p* < 0.001. The estimated spans for the 2468 list type amounted to 7.98 for the memory span and 7.59 for the task span. With the 2369 list type, the spans were 7.74 and 7.36 respectively. These values are higher than the ones observed by Logan. One possibility is that human subjects probably do not use chunking all the time because of the large mental effort involved.

### Three-item chunks: Results and Discussion

When the simulation attempted to form 3-term chunks all the time, the results were not much higher than those obtained with 2-item chunks. The results are shown in the bottom panel of ***Table 9***. First, consider the results with the 2468 list type. The proportions decreased over list length, *F*(3,26) = 389.03, *p* < 0.001, *η_p_*^2^ = 0.98, this trend was linear, *F*(1,28) = 736.63, *p* < 0.001, *η_p_*^2^ = 0.96 (87% of the variance). The proportions were overall larger for the memory span than for the task span (respectively 0.83 and 0.80), *F*(1,28) = 8.40, *p* < 0.01, *η_p_*^2^ = 0.23. List length interacted with Span type, *F*(3,26) = 17.95, *p* < 0.001, *η_p_*^2^ = 0.67, as did the linear trend over list length, *F*(1,28) = 5.52, *p* < 0.05, *η_p_*^2^ = 0.17.

Also for the 2369 list type, proportion recalled decreased over list length, *F*(3,26) = 154.11, *p* < 0.001, *η_p_*^2^ = 0.95, and did so linearly, *F*(1,28) = 478.10, *p* < 0.001, *η_p_*^2^ = 0.95 (86% of the variance). Task span was larger (M = 0.87) than memory span (M = 0.81), *F*(1,28) = 20.27, *p* < 0.001, *η_p_*^2^ = 0.42, and interacted with list length, *F*(3,26) = 7.43, *p* < 0.001, *η_p_*^2^ = 0.46; linear trend, *F*(1,28) = 11.98, *p* < 0.01, *η_p_*^2^ = 0.30.

The observed and chunk-3 proportions correlated 0.90, *t*(30) = 11.13, *p* < 0.001. In the 2468 list type the memory span was 8.0 and the task span 7.8. In the 2369 list type, the estimated values were respectively 8.90 and 9.0.

### Aggregation of results

The high values for memory and task span observed under conditions with 2 and 3-term chunking suggest that the values observed by Logan (2004) quite likely involve occasional chunking. Assuming that it would be convenient to form 3-term chunks when the list lengths are predominantly multiples of three and 2-term chunks when the list lengths are only multiples of 2, and furthermore taking into account that working with chunks creates an advantage but that this comes with the cost of a lot of effort, a reasonable estimate could be that such chunking is attempted in about one half of the occasions, avoiding chunking at all in the other half. Based on this rationale the following exercise was performed. The proportions obtained in the 2468 list type in the no-chunking application and the 2-term chunking application were averaged. Similarly, for the 2369 list type the no chunking proportions and the 3-term chunking proportions were averaged. On the basis of these averaged proportions the memory and task span were estimated. These averaged proportions and the observed proportions correlated 0.95, *t*(30) = 16.26, *p* < 0.001. The resulting span estimates are shown in ***Table 10*** in the row average, and suggest that the proportion of chunking in the second list type is underestimated.

**Table 10:** Observed and estimated memory and task spans based on a weighted combination of different degrees of chunking in the two list type conditions.


SPAN	LIST TYPE 2468		LIST TYPE 2369	
	
	MEMORY	TASK	MEMORY	TASK

Data	6.58	6.14	7.25	6.64

Average	6.46	6.36	6.83	6.99

Estimate	6.52	6.49	6.80	6.92


Alternatively, the weights for each of the three simulation outcomes, no chunking, chunks of two and chunks of three can be estimated to obtain a best fit to Logan’s data. The best least squares fit was obtained with respective weights of 0.48, 0.09, and 0.425. The estimates and the original data reported by Logan (2004) are shown in ***Table 10***. These simulated spans are very similar to the spans reported by Logan, although the difference between memory and task spans tends to be even smaller in the simulations.

All these findings regarding the task span taken together, it can be concluded that the WMDEC model accounts for Logan’s 2004 observations and it can do so because the load on working memory for memorisation is mainly on the episodic buffer while task execution taxes predominantly the executive module with intermittently brief and low loads on the episodic buffer.

## STUDY 5. COMBINATION OF TASK SWITCHING AND DUAL-TASKING

From the viewpoint of working memory research, the findings reported by Logan (2004) were quite surprising. One key feature in these experiments is that subjects performed the task without any time constraints: there were no response deadlines neither for task execution nor for recall. From the viewpoint of the Time-Based Resource Sharing (TBRS) account of working memory (e.g., Barrouillet et al., 2004; Barrouillet & Camos, 2020), dual-task costs in working memory are caused by time constraints. If there are no time constraints, one can simply take the time to refresh the working memory contents and put the secondary task on hold for a while, and it was suspected that this may be a key element in Logan’s results.

Based on this rationale, Liefooghe et al. (2008) designed a series of experiments to test the dual-task effects of task switching in a recall context with strict timing of the events, as prescribed by the logic behind the TBRS view. Subjects were presented with a sequence of letters for recall and in the inter-letter and/or in the retention interval series of digits were shown for magnitude or parity categorisation. The frequency with which these two digit categorisation tasks switched was varied, while everything else was the same across conditions. In brief, these experiments confirmed that short-term serial recall was poorer when more frequent switches between the embedded secondary tasks was required. In order to demonstrate that WMDEC not only accounts for Logan’s findings but also for the time-constrained findings, the computational version of WMDEC was applied to the experiments of Liefooghe et al.

### Dual-task effects of repetitions and alternations

In their first experiment, Liefooghe et al. (2008) compared short-term serial recall of lists of 3 to 6 letters, while after the presentation of each letter 8 digit decision tasks were performed in the list procedure. The tasks used were magnitude and parity judgment. The task lists were either single-task lists (8 magnitude tasks or 8 parity tasks) or alternating-task lists (magnitude and parity in alternation, either starting with magnitude or starting with parity). Each digit in the lists was either shown in red (magnitude) or in blue (parity), so that colour could be used as a cue. As is typical in such task switching experiments with the list procedure (Allport et al., 1994; Jersild, 1927; Spector & Biederman, 1976), the responses were slower in the alternating as compared to the single-task lists. As a consequence, according to the TBRS model, central attention was occupied for a longer duration in the alternating lists than in the single-task lists, and this should and did result in poorer recall.

In earlier sections of this article, the computational version of WMDEC was already applied to this type of dual-task situation, so that in principle, this application was straightforward. However, it should be noted that this setting can be considered as a triple-task setting: apart from the memorisation and recall task, in the maintenance interval two tasks alternated at a fast rate, so that it was commendable to keep the two alternating tasks in working memory so as to avoid long periods of task-set reconfiguration, especially as the inter-digit interval was limited to 1.2 s. Because of this strict and fast timing, it was decided to set the response threshold in WMDEC to a lower level than in the other applications.

As explained in an earlier section, in WMDEC the response threshold varies during an experimental session on the basis of explicit or implicit feedback: consecutive correct responses lead to lowering the threshold. In the present application, it was assumed that subjects in such a time-constrained experimental setting would decide to lower the response threshold in order to save time for refreshment of memory contents. Thirty-two statistical subjects were involved in this simulation.

#### Results

Liefooghe et al. (2008) calculated proportions of correct recall to have a measure that is more comparable across different list lengths. ***Table 11*** displays the observed and simulated results for correct recall in absolute position. In the simulation, the proportion of correctly recalled letters was larger in the single-task (0.95) than in the alternating-task lists (0.78), *F*(1,31) = 98.41, *p* < 0.001, *η_p_*^2^ = 0.76. The recall proportions decreased with list length as is displayed in the top panel of ***Table 11***, *F*(3,29) = 13.49, *p* < 0.001, *η_p_*^2^ = 0.58. The correlation between the simulated and the observed proportions correct recalls amounted to 0.86, *t*(6) = 4.14, *p* < 0.01.

**Table 11:** Average proportion correct recall in position in the three experiments of Liefooghe et al. (2008) and in the WMDEC simulations of these experiments.


		LIST LENGTH					

		3	4	5	6	7	8

Experiment 1							

Observed	Single	0.96	0.96	0.89	0.79		

Observed	Dual	0.82	0.88	0.83	0.74		

Simulated	Single	0.99	0.98	0.95	0.88		

Simulated	Dual	0.81	0.81	0.83	0.70		

Experiment 2							

Observed	Few	0.86	0.83	0.79	0.71		

Observed	Many	0.85	0.79	0.70	0.68		

Simulated	Few	1.00	0.92	0.90	0.85		

Simulated	Many	0.90	0.88	0.87	0.80		

Experiment 3							

Observed	Few	0.94	0.91	0.84	0.71	0.70	0.60

Observed	Many	0.90	0.90	0.81	0.67	0.72	0.49

Simulated	Few	0.89	0.74	0.68	0.63	0.56	0.49

Simulated	Many	0.87	0.72	0.67	0.57	0.54	0.47


### Effects of switch frequency

In their second experiment, Liefooghe et al. (2008) compared short-term serial recall of 3–6 letter lists when the task lists contained few (2–3) or many (5–6) task switches. Otherwise, the procedure was completely similar to the first experiment.

#### Results

The results of the application of the computational version of WMDEC is shown in the central panel of ***Table 11***, and revealed that proportions of correct letter recalls in absolute position were higher when the task lists contained few (0.92) rather than many (0.86) switches, *F*(1,31) = 11.47, *p* < 0.01, *η_p_*^2^ = 0.27, and also decreased with list length, *F*(3,29) = 17.45, *p* < 0.001, *η_p_*^2^ = 0.64. The correlation between these simulated and observed recall proportions amounted to 0.87, *t*(6) = 4.25, *p* < 0.001.

### Switch frequency with task switching in the retention interval

The third experiment of Liefooghe et al. (2008) used memory lists with lengths from 3 to 8 consonants; the tasks were executed after all letters had been presented (in the retention interval) as four lists of 8 tasks (Brown-Peterson paradigm). In all other respects, the settings were the same as in the previous experiments.

#### Results

The observed and simulated proportions correct recalls are shown in the bottom panel of ***Table 11***. In the simulation, proportion of recall differed among the number of switch conditions, *F*(3,29) = 4.76, *p* < 0.01, *η_p_*^2^ = 0.33, but was not significantly higher when there were fewer switches (0.67 v. 0.65), *F*(1,31) = 2.08, *p* = 0.159 *η_p_*^2^ = 0.06. Proportion of correct recalls decreased with list length, *F*(5,27) = 86.94, *p* < 0.001, *η_p_*^2^ = 0.94. The correlations of these simulated proportions with the observed proportions was 0.90, *t*(10) = 6.54, *p* < 0.001.

The correspondence between simulated and observed data was excellent, yet the proportions correct recalls tended to be lower than the observed proportions.

### Discussion

The correspondence between the observed and simulated correct recall proportions is very close. This can be seen when inspecting these values in ***Table 11*** and in the quite high and significant correlations between these two sets of proportions. Besides, the important effect of the frequency contrast (all-or-none in the first experiment; many-or-few in the other experiments) was confirmed: recall was poorer when more switches occurred. Switches occupy task processing for a longer time, postponing refreshment actions on the stored letters for a longer time, so that a larger loss of the memory traces occurs under the conditions where fewer refreshments are possible.

Like the observed data, the simulations also showed that proportion of correct recall decreased with length of the memory lists. This effect is not specific to a dual-task context but it is a general effect: as the lists become longer less time is available to refresh each individual to-be-recalled item and as the load on working memory increases with the number of to-be-recalled items, some memory loss is bound to occur which results in poorer recall.

It may be argued that the fact that the WMDEC model accounts as well for the data reported by Logan (2004) and the data of Liefooghe et al. (2008) shows that the model cannot be consistent as it accounts for opposing findings. It would indeed be problematic if the model would account for the two opposing sets of results on the basis of the same involved mechanisms. However, it must be pointed out that the reason why WMDEC accounts for the Liefooghe et al. findings is related to the fact that under strictly timed conditions, the amount of time to refresh memory elements is limited, which results in memory losses leading to poorer recall. The reason why the WMDEC also accounts for Logan’s findings resides in the fact that in order to perform the tasks while maintaining the names of these tasks, two different working memory modules are involved so that while the tasks are being performed the task names can be maintained. Moreover, because there are no time-constrains in executing the tasks, refreshment remains possible.

## GENERAL DISCUSSION

The major findings of these simulation studies based on the WMDEC model can be summarised as follows.

1. The model accounts for the performance costs associated with task switching, with shorter preparation time, and for the reduction of the switch cost with longer advance preparation time. Besides, although the switch cost decreased with longer preparation time, even at the longest preparation intervals, a substantial residual switch cost remained. These findings are observed in response time and and were confirmed in Studies 1 and 2; in accuracy the observations were less clearcut but roughly followed the same pattern.
2. Furthermore, under the assumption that orientation or dimension of attention is a task set parameter, the model accounts for the findings regarding a larger cost associated with task switching than with dimension switching (e.g. Kleinsorge & Heuer, 1999; Kleinsorge et al., 2001, 2004), but under the assumption that task and dimension changes require dedicated combined task sets, the model accounts for the findings supporting a so-called flat structure (e.g., Allport et al., 1994; Vandierendonck et al., 2008).
3. With respect to dual-tasking, the model simulations show larger memory loss under higher cognitive load, as for example in experiments reported by Barrouillet and colleagues (e.g., Barrouillet et al., 2007).
4. In the context of dual-tasking, the model simulations also fit the findings of very small differences in estimated memory and task span as reported by Logan (2004). The reason the model fits these data is due to the fact that support for task execution and memory performance comes from two separate WMDEC modules, namely EB and EM, and that the tasks are performed at leisure so that task execution can be managed to leave room for memory refreshment.
5. Finally, in situations where dual-task coordination is required during memorisation (in between the presentation of successive recall elements) or during the retention interval, the more task switches are required during the maintenance and retention interval, the larger the memory loss (e.g., Liefooghe et al., 2008). Simulations of the model also fit these findings and these effects are due to the amount of time that remains available for refreshment of the to-be-recalled materials.

All the predicted effects were significant in the simulation data and in all five studies the correlations between the observed and the simulated means of the relevant dependent variables were large and significant, except for the span length in Study 3, where the significance was attained but not crossed.

As the results in all five simulation studies were consistent with the expected/predicted findings, it may be concluded that the model accounts for these findings. Clearly, this model is not the only computational model that accounts for the findings regarding preparation and residual switch costs in task switching (Studies 1 and 2). Several computational models of task switching have been published in the last decades. Although some models, such as the model of Gilbert and Shallice (2002) address specific issues regarding interference and task set inertia, the models of Reynolds, Braver, Brown and Van der stigchel (2006) and Brown, Reynolds and Braver (2007) account for all these effects, and the same goes for the model of Altmann and Gray (2008) that integrates episodic memory and task performance and control phenomena.

For an account of the phenomena addressed partly in Study 2 and fully in Studies 3–5 it does not suffice to call on a model that accounts only for task switching phenomena. Studies 3–5 combine task performance with working memory and in order to properly account for these combined phenomena an extended working memory model is needed. In principle, a computational version of Oberauer (2009)’s design for working memory could account for such phenomena, but to my knowledge this has not been attempted thus far. Oberauer and Lewandowsky (2011)’s computational version of the TBRS model of Barrouillet and Camos (2020) was applied to complex span situations and to the research by Liefooghe et al. (2008) and so this model can account for Studies 3 and 5, but not Studies 1 and 2 and quite likely not Study 4 because these findings are not consistent with expectations from the TBRS view. Similarly, the computational version of TBRS in the model of Glavan and Houpt (2019) uses the experiment of Study 3 as a benchmark and hence accounts for Study 3, but one can only guess whether the model would also account for Study 5. Again, because Studies 1, 2 and 4 are outside the scope of the model, there is no evidence that this model could account for those data.

One more model deserves to be mentioned in this context, namely the ‘Threaded cognition’ model of Salvucci and Taatgen (2008). This model, developed within the ACT-R architecture (Anderson et al., 2004), is proposed as an integrated theory of multi-tasking. In threaded cognition, streams of thought or action are represented as threads that connect the required resources. In a multi-tasking context, several overlapping threads are active. When one of the resources (perception, motor system, memory, …) is busy to another thread, delays are expected to occur. Occasionally, several threads will compete for a particular resource, in which case a conflict resolution mechanism will decide which thread gets priority. The usage of threads is similar to the goal and task-set representation connections in WMDEC, but the threaded cognition model is more general than WMDEC, so that the theory is able to handle a much broader scope of multi-tasking situations than presently considered in the computational version of WMDEC. Notwithstanding this broader scope in the threaded cognition view, WMDEC has a few advantages. First, it comes with an elaborated view on working memory allowing for predictions about memory performance under load. Second, WMDEC also considers competition between sequential threads (as in task switching) as a factor that may introduce processing delays, whereas in threaded cognition, task switching is considered as a single thread with as a consequence that the model does not yield a straightforward account of switching costs. The latter implies that it would be difficult for the threaded cognition model to account for the present findings with respect to task-switching (Studies 1, 2, 4, and 5), whereas more development is needed to achieve a computational version of WMDEC that accounts for a number of multi-tasking effects such as, for example, overlapping driving and dialling.

In sum, it appears that the computational version of WMDEC is the only one of all these models that accounts for all the data used in the present simulation studies. That the model accounts for such a broad range of findings, testifies to the adequacy of the model in its combination of temporary memory storage and the actions required to prolong such storage on the one hand, and temporary storage for actions that relate to goal-directed activities, on the other hand. For sure, there are many more tests needed before one can be confident about the validity of the entire model. Nevertheless, the observation that the model accounts for this combination of findings is interesting, in particular because it seems to be the only working memory model that can account for this range of findings in appropriate formal tests of the model.

Notwithstanding this rather positive appreciation, some concerns may be raised. One important concern relates to the fact that small changes in the assumptions as laid down in the condition-action rules may lead to different predictions. First thing to be said in this respect, is that working with different condition-action rules is similar to changing the model parameters. The condition-action rules constitute a large set of degrees of freedom that can be used to enhance the predictions of the model. It should be stressed, though, that this was not the strategy followed in the present simulations. In particular, in the simulations regarding task and dimension switching, the same condition-action rules were used as in the other simulations with magnitude and parity tasks, and on the basis of this set of rules, findings as those reported by Kleinsorge and colleagues (e.g., Kleinsorge et al., 2002) were predicted. Only as a next step, the question was raised whether with an appropriate but limited change in the assumptions (i.e., condition-action rules) the model could account for the other findings in the literature. Interestingly, it did.

In these comparisons, the model as programmed is the same, but the condition-action rules reflect other learning experiences and/or other decisions about how to use such previous experience. Basically, this small difference in condition-action rules accounts for obtaining a hierarchical or a flat structure in the task-set organisation. In other words, if we can observe that under some circumstances some humans prefer to manage a situation in a particular way, while the same or other persons in different situations prefer to manage the situation in another way, we would be inclined to suggest that humans show flexibility in their management of difficult situations. In a similar way, it can be suggested that the model, and in particular the selected condition-action rules, provide a possible account of such cognitive flexibility. And, as always in scientific endeavour, this conclusion is not the final answer, but should be tested by novel experimentation.

In a similar vein, the last set of simulations used a small change in quantitative parameter values of the model. At the outset, the intention was to use exactly the same quantitative parameter values in all the applications of the model. And this was indeed the case, except for the simulations of the Liefooghe et al. (2008) findings. In these simulations, the parameter for the response threshold was set to a lower level, so as to produce faster but possibly less accurate responses. It is a basic property of the model (see Vandierendonck, 2020) that it adapts the speed-accuracy balance on the basis of the response conflicts (Botvinick et al., 2001) and monitoring of the correctness of the emitted responses, so that after many correct responses, the response threshold is lowered while after errors, the response threshold is raised. As it is implemented in the model, the range of changes made is rather limited. A more extensive test and calibration of this mechanism is needed to ensure that this specific part of the model forms a good representation of such reactive control actions in the way they occur in many situations (e.g., Gratton, Coles & Donchin, 1992) Apparently, the calibration of the speed-accuracy control in the model can be improved, as it appeared that without a lower response threshold, many of the responses were emitted too late leaving no time for memory refreshment, so that a parameter adaptation was useful to perform a proper test of the model.

An important concern with models as complex as WMDEC relates to the question whether the model is not too complex and too flexible at the expense of parsimony. This may be tested by deleting some part of the model and check whether it still works. Performing a blunt test by deleting for example the executive memory module cannot be very informative, because when this module is deleted, the entire mechanism allowing for adaptive goal-directed behaviour is corrupted. So, indeed if this part of the model is deleted, none of the predictions tested in the present paper would be borne out. Similarly, deletion of the episodic buffer would mutilate the core of working memory storage and would result in failure in all simulations. Note however that although the model is equipped with a phonological loop and a visuo-spatial module, neither of these were of any use in the present simulations. They were not deleted from the model, but they were not used because no production rules involving these modules matched the conditions. Instead of deleting parts of the model, more subtle tests of the model’s complexity/parsimony balance can be performed. One way to achieve this is by setting a targeted model parameter to a limiting value (0 or 1, for example), and see how the predictions are affected. A second possibility consists of deleting some of the production rules to see whether the predictions would still hold. As the present article is already quite long as it is, no attempts were reported to test modelling limits, but it would indeed be interesting to explore the effects of extreme parameter values and/or deletion of some production rules.

The question may also be raised whether the model predicts any new phenomena. In comparison to other working memory models, it may be pointed out that WMDEC extends the set of predicted phenomena outside the working memory realm. However, because the model was developed with the focus of doing so, it is more interesting to check whether there is anything really new, i.e., something that has not yet been predicted. One partly new and two completely new predictions can be be mentioned here.

1. First, because the working memory system consists of three storage units (episodic buffer, phonological loop, and visuo-spatial module) each with its own maintenance preservation mechanism (attentional refreshment for EB, rehearsal for PL and revival for VSM), it follows that maintenance and recall can be based on one, two or even all three storage units. Considering that recall always involves the episodic buffer (with or without refreshment), it follows that when both refreshment and rehearsal have been used, recall performance will be better than when only one of both has been applied. Similarly, for recalls that involve visual, spatial or location information, recall will be better when refreshment and revival have been used than when only one of both is active (this is similar to predictions by Barrouillet & Camos, 2020). In fact, three specific predictions can be formulated: (a) the longer the sequence of elements to be recalled in correct order, the more recall based on refreshment only will be superior over recall based on rehearsal only or on revival only; (b) recall based on a combination of refreshment and rehearsal will be larger than recall based on either refreshment or rehearsal alone, but it will not be as large as their sum; (c) similarly, recall based on a combination of refreshment and revival will be larger then recall based on either refreshment or revival, but it will not be as large as their sum.
2. As WMDEC considers memorisation and recall as goal-directed activities, and that retrieval requires the activation of a specific action, it follows that when the required action must be changed during the retrieval process, a cost is incurred such that retrieval performance will be poorer with than without such a change. More specifically, consider a situation in which retrieval starts with serial recall and that after some time a signal indicates that retrieval must continue by a recognition procedure, it will take some time to change the ‘mind set’ (i.e., to replace the action) before retrieval can continue. In the meantime, the memory traces may have weakened, wich is a direct cause for poorer retrieval performance. This prediction concerns as well shifts from recall to recognition as from recognition to recall.
3. Consider a task such as the so-called ‘double-span task’ (e.g., Martein, Kemps & Vandierendonck, 1999), in which words or pictures are presented at particular locations in a matrix with the instruction to remember both the identity of the presented elements and their location in the order of presentation. At retrieval, one is told to recall both identity and location or only one of both. According to WMDEC this retrieval instruction results in activation of the appropriate recall action. If after having recalled two of the items, the subject is signalled to switch to another retrieval mode (e.g., from identity to location, or vice versa), this would require a shift to another retrieval action. This is expected to result in a ‘retrieval switch cost’, such that recall performance is expected to be poorer with such a switch than without switch.

Taken all together, the results obtained in these simulations of the model are coherent and show how the model is able to combine memory and action representations in task switching and dual-tasking. These simulations show clearly that while in task switching, competition driven by lateral inhibition between the task goals and task sets is at the basis of the observed effects, in a dual-task context such a competition would be rather harmful, and indeed dual-tasking can only be successful if the goals and task sets can to some extent exist together and operate simultaneously in working memory. As no other forms of multi-tasking apart from task switching and dual-tasking have been considered, the findings obtained with the WMDEC model cannot be generalised to these other contexts. Nevertheless, as a more general conclusion of the present work, it may be said that the present findings suggest that a limited set of control actions governed by condition-action rules that specify strict conditions under which the control actions are taking place may account for multi-tasking generally. In order to sustain this conclusion, however, more (simulation) research will be needed.

## DATA ACCESSIBILITY STATEMENT

The software to run the computional implementation of WMDEC described in this article and the raw simulation results are available at *https://doi.org/10.5281/zenodo.3997259*.

## ADDITIONAL FILES

The additional files for this article can be found as follows:

10.5334/joc.138.s1 Appendix A. Illustration of dLTM contents.

10.5334/joc.138.s2 Appendix B. Condition-action elements.

## Footnotes

The duration of the cycle is chosen for computational convenience: on the one hand, when the cycles are shorter, more precision can be achieved in modelling the processes. As an example, consider a random walk approach to Ratcliff (1978)’s diffusion model: the discrete steps in the random walk must be very short to attain an acceptable approach to the continuity assumed in the model (0.05 ms according to Tuerlinckx, Maris, Ratcliff & De Boeck, 2001). On the other hand, the shorter the cycles the longer the time needed for running a simulation of the model. The 10-ms choice has a few advantages: execution by the computer is reasonably fast even if parameter estimation procedures must be run, and it is sufficiently accurate. Moreover, multiplying the model’s response time by 10 yields time estimates that can be directly compared to ms response times by human subjects.

For clarity, the symbols used in the computational model are shown in type writer print.

The notation c62 refers to cycle 62.

It can be argued that a better test is achieved by fitting the model parameters. However, the type of model tested here has so many parameters that closer fits are indeed possible. It should be pointed out, though, that a test based on fitted parameters is not necessarily more convincing than the type of test performed here. For one thing, some of the processes modeled are only approximations. For instance, stimulus identification in the task and dimension switching is such an approximation and the identification time is likely an underestimation. As a consequence parameter fitting would then result in overestimation of the other processing stages. It can be doubted that a fit obtained under those conditions is more valuable than the correspondence obtained with the present procedures. Because the predictions in majority concern effects of experimental manipulations, the simulated data were analysed for the presence of these effects. Because the variance within simulations tends to be smaller than the variance of the data, the absolute size of the effects in the simulations should be treated with care.

Note that with this implementation, no assessment of the number of elements is required so that it is likely that stimulus processing in the simulation could be faster than in the experimental situation.
