# Supplementary material for: How the Working Memory with Distributed Executive Control Model Accounts for Task Switching and Dual-Task Coordination Costs
Source: J Cogn. 2021 Jan 7;4(1):2. doi: 10.5334/joc.138 (PMC7792467; doi:10.5334/joc.138)
Supplement: Appendix A. — Illustration of dLTM contents. [file joc-4-1-138-s1.pdf]

Appendix A

Illustration of dLTM contents

Within WMDEC, dLTM is conceptualised as an associative network with different types of associations or links. Apart from links that constitute a hierarchical structure, superordinate (`sup`) and subordinate (`sub`), also property links (`has`), opposites (`isop`) and sequence links (`next`) are included. In the computational implementation, the network includes internal symbols as well as external ones. The external symbols refer to entities or events that exist in the surrounding world. For example, in a simulation of a memorisation experiment requiring recall of letters, the letters are represented in dLTM with symbol names `WA-WZ`. They are at the lowest level of the hierarchy and have the symbol `LETTER` as superordinate link and they have properties such as `VOWEL` or `CONS`[6]. Other objects used can be digits (`D0-D9`) with superordinate `DIGIT`, or shapes (`CIRCLE, TRIANG, SQUARE, DIAMOND, CROSS, ...`). Objects that are used in a memorisation task in addition have the property `TARGET`. Some stimuli are used as cues (e.g., `CMEM, CRCL, CMAG, ...` to a memorisation, a recall or a magnitude judgment task), they have the superordinate `CUE` and they can have properties (e.g., the `CRCL` may have the property `ORDER` to indicate that order recall is required). An example of these objects and their links is displayed in Table A1.

Internal symbols are mainly used to refer to representations in working memory that are representing objects or events originating from within the WM system (see Table A2, for a subset of these symbols). A straightforward example is `GOAL`; for example, memorisation (`MEM`), and magnitude judgment (`MAG`). These goals are instantiated in EB and when several goals are present, the one with the highest level of activation is dominant and constrains other processes within WM. In order to be effective, the goal must be supported by a task set (`TSKSET`) which is a structure instantiated in EM. Examples of task sets are `MEMTS` to support memorisation, recall, recognition, refreshment, rehearsal, etc. and `MAGTS` to support the magnitude goal. The operation of the task set depends on its components. Every task set contains a list of parameters. For a memorisation task set these parameters include `MMOD` (memory modality: oral or visuospatial), `RMOD` (response modality: oral, manual), `MNTAIN` (how to maintain information), `RCL` (recall `OFF` for memorisation, `ON` when recall is required),

[6] `CONS` is an abbreviation of ‘consonant’. Most of the symbols are abbreviations or contractions of existing words and these short forms are used for convenience.

CHSIZE (maximal chunk size, typically L0, L2 or L3), and DREF (refreshment schedule: ALL, F1–F3, or L1–L3, i.e., refresh all, the first 1, 2 or 3 or the last 1, 2, or 3). The maintenance parameter can take of a series of values: refresh EB (RF), rehearse PL (RH), revive VSM (RV), a combination of these (RFH, RFV, RHV, RFHV), or do nothing (NORFHV). A memory task set also includes one or more possible actions, namely refresh (RFRSH)[7], and retrieval (RTRIEV). Other types of task sets such as for example magnitude judgment has a few task parameters, namely response modality (RMOD), binding control (MKBIND which is ON or OFF), response control (RSPCTL with values BLOCKED, UNBLOCK and DONE), and possibly also stimulus or attenttional dimension (SDIM). Such a task set also typically has a set of category-response mappings (e.g., SMALL-LEFT, LARGE-RIGHT).

Clearly, internal symbols are not only used to refer to goals and task sets, but also to their parameters, mappings and actions, and even to the values of the parameters, etc. Besides, internal symbols are also used to distinguish different types of representations: apart from goals and task sets, objects stored in WM have a type that corresponds to different ways of processing, such as a binding of several entities (BIND), a chunking (CHUNK), a goal coordination (COORD), a category (CLASS), a response label (RESPONSE), or a feedback (FDBCK). Not all of these symbols are shown in Table A2 but only a subset so as to give an idea of the symbols and their organisation in dLTM.

[7] Refreshing is assumed to be a controlled activity requiring attention and effort, while rehearsal and revival once initiated run off automatically and do not require task set control.

Table A1

Example of dLTM contents: External symbols. Different types of links between dLTM entries are shown: superordinate (sup), subordinate (sub), property (has), opposites (isop) and sequential (next). In order to keep the example clear, only a few of the lowest level entries are shown (letters, digits, shapes).

Entry	sup	sub	has	isop	next
EXTL	–	CUE OBJECT			
CUE	EXTL	CMEM CRCL			
OBJECT	EXTL	DIGIT WORD SHAPE			
SHAPE	OBJECT	CIRCLE SQUARE ... CROSS	TARGET		
CMEM	CUE		MEM RFRSH RHRS RVV WORD	CRCL	
CRCL	CUE		MEM RECL ORDER WORD	CMEM	
DIGIT	OBJECT	D0 D1 ... D9			
LETTER	OBJECT	WA WB ... WZ	TARGET		
TARGET	EXTL				
WA	LETTER		VOWEL		WB
WB	LETTER		CONS		WC
WZ	LETTER		CONS		
D0	DIGIT		DIG		D1
D1	DIGIT		DIG		D2
D9	DIGIT		DIG		
CIRCLE	SHAPE				
SQUARE	SHAPE				
CROSS	SHAPE				

Table A2

Example of dLTM contents: Internal symbols. These are symbols that refer to internally generated objects, parameters, actions, properties, ... that are used to build representations in working memory. The table does not contain the complete list, but only an exemplary subset.

Entry	sup	sub	has	isop	next
INTL	–	GOAL COORD RESPONSE ... FDBCK TSKSET ACTION			
GOAL	INTL	MEM PAR MAG			
MEM	GOAL		CMEM CRCL		MEMTS
MAG	GOAL		CMAG	PAR	MAGTS
COORD	INTL	DUAL	MEM PAR MAG		
RESPONSE	INTL				
FDBCK	INTL				
TSKSET	INTL	MEMTS TASKTS			
ACTION	INTL	RHRS RVV			
RHRS	ACTION		RH ... RFHV		
RVV	ACTION		RV ... RFHV		
MEMTS	TSKSET	MEMPAR MEMACT	MNTN RTRIEV		
MEMPAR	MEMTS		MMOD ... RCL		
MMOD	TSPAR		ORAL VS		
RCL	TSPAR		OFF ON		
MEMACT	MEMTS		MNTN RTRIEV		
MNTN			RF ... RFHV		
RTRIEV			RECL RECO		
TASKTS	TSKSET	MAGTS PARTS			
MAGTS	TASKTS	MAGPAR MAGMAP	PARTS		
MAGPAR	MAGTS		RMOD ... RSPCTL		
MAGMAP	MAGTS		SMALL LARGE		
RMOD	TSPAR		NONE MANUAL ORAL		
RSPCTL	TSPAR		BLOCKED UNBLOCK DONE		
TSPAR		MMOD ... RCL			
