# Supplementary material for: How the Working Memory with Distributed Executive Control Model Accounts for Task Switching and Dual-Task Coordination Costs
Source: J Cogn. 2021 Jan 7;4(1):2. doi: 10.5334/joc.138 (PMC7792467; doi:10.5334/joc.138)
Supplement: Appendix B. — Condition-action elements. [file joc-4-1-138-s2.pdf]

Appendix B

Condition-action elements

Table B1 shows the Backus Naur Form syntax definition of the subset of the production rules that are relevant for the present simulations. The function-like forms such as 'action(...), actmode(...), etc.' are defined in Tables B2 and B3. On each cycle of the procedural loop, all the production rules, except the already active ones, are compared to the contents of the memory system. To check whether a production rule matches the current status of the system, each condition element of the rule condition is compared to the memory contents; this continues until either one of the elements does not match (non-match of the rule) or all the elements in the condition match memory contents (match of the rule). Rules that match are added to the list of matching rules. When all rules have been checked, one of the new matching rules is selected for execution; the other rules are removed from the list. A rule that matches may become immediately active or may have to wait for execution if the targeted object is occupied by another active rule. At the time of selection, it is noted for how many cycles the rule will remain active; this depends on the minimal duration as specified in the rule and may be adapted depending on operational conditions.

Table B1

Syntax definition for the set of production rules used in the present applications of the model. The list of the allowable symbols is open ended as new ones can be added as long as they are defined in the declarative LTM module.

<rule> :: IF <condition> THEN <action>

<condition> :: <conelement> | <conelement> AND <condition>

<conelement> :: <conspec> | !<conspec> | <var> = <conspec> | <conspec> <cmp> <arg>

<conspec> :: action(<arglist3>) | actmode(<arg>) | chpart(<arg>) | crmap(<arglist2>) | domgoal() | domts() | gamsget(<arg>) | getage(<arg>) | has(<arglist3>) | ineb(<arglist5>) | inem(<arg>) | instr(<arglist2>) | isnew(<arglist2>) | location(<arglist2>) | ltmhas(<arglist2>) | ltmlink(<arglist2>) | ltmnext(<arg>) | ltmsup(<arglist2>) | nbind(<arg>) | newcnt(<arg> | param(<arglist2>) | parstr(<arglist2>) | str(<arglist3>) | tskinstr(<arglist2>)

<arg> :: <var> | <symbol>

<var> :: V, W, X, Y, Z

<cmp> :: == | != | > | >= | < | <=

<arglist2> :: <arg>,<arg>

<arglist3> :: <arg>,<arg>,<arg>

<arglist4> :: <arg>,<arg>,<arg>,<arg>

<arglist5> :: <arg>,<arg>,<arg>,<arg>,<arg>

<action> :: addbind(<arglist2>) | categorise(<arglist2>) | compbind(<arglist2>) | defact(<arglist3>) | defmap(<arglist3>) | do(<arg>) | execute(<arglist2>) | growts(<arg>) | incstr(<arglist2>) | learn(<arglist3>) | loadgoal(<arg>) | mkbind(<arglist2>) | mkchunk(<arglist2>) | mkinst(<arglist3>) | mktbr(<arglist4>) | mkts(<arg>) | refresh(<arglist2>) | rehinit() | release(<arg>) | retrieve(<arg>) | revinit() | respond(<arg>) | setgams(<arglist2>) | setmode(<arglist2>) | setpar(<arglist3>) | unbind(<arg>)

<symbol> :: NONE, OFF, ON, EMPTY, ANY, ...

Table B2

Condition elements used in the set of production rules in the present simulations. The left column shows the name and the arguments of the condition element, the second column indicates the module addressed, and the final column gives a short explanation of the meaning.

Condition	Use	Explanation
action(ts,a,p)	EM	property p (status or target) of action a of task set ts
actmode(ts)	EM	name of currently active action of task set ts
chpart(o)	EB	next recallable part of chunk o
crmap(ts,class)	EM	response of category-response map for class in task set ts
domgoal()	EB	goal object in EB with highest level of activation
domts()	EM	task set in EM with highest level of activation
gamsget(par)	GEN	value of parameter par in general attention/motor system
getage(o)	EB	age (elapsed cycles since creation) of object o
has(o,f,value)	VSM	value of feature f (colour, position, ...) of visual object o
ineb(a,b,c,loc,lev)	EB	TRUE if object a of type b with property c exists in EB on location loc and is accessible (if lev = 1)
inem(ts)	EM	TRUE if ts exists in EM
instr(ts,par)	GEN	instructed value for parameter par of ts
isnew(sm,i)	SM	the ith new element from sensory memory sm
location(var,i)	VSM	location of ith new element with name var
ltmhas(a,b)	DLTM	TRUE if object a has a property link to prop
ltmlink(a,b)	DLTM	return object name of object linked to a and has property b
ltmnext(goal)	DLTM	task set corresponding to goal object
ltmsup(a,b)	DLTM	TRUE if b is superordinate of a
nbind(o)	EB	number of elements bound by object o
newcnt(sm)	SM	number of new elements in sensory memory sm
param(ts,par)	EM	value of task set parameter par of ts
parstr(ts,par)	EM	strength of task set parameter par of ts
str(mod,t,o)	GEN	activation of object o of type t in module mod (EB or EM)
tskinstr(cue,p)	GEN	TRUE if instruction links cue to property p

The named module abbreviations in the second column are the following: EB (episodic buffer), EM (executive memory), VSM (visuospatial memory), DLTM (declarative LTM), SM (sensory memory, either iconic or echoic), GEN (general or more than one module involved).

Table B3

Actions used in the production rules of the present simulations. The first column specifies the action including its arguments, the second column specifies the addressed module, and the third column contains a brief explanation.

Action	Use	Explanation
addbind(tgt,class)	EB	add class to bind tgt
categorise(o,cat)	EB	assign category cat to object o
compbind(tgt,resp)	EB	add response so as to complete bind tgt
defact(ts,pnam,pval)	EM	set up task action pnam with value pval in ts
defmap(ts,par,val)	EM	set up task set mapping for par with value val
do(val)	GEN	activate response (go with flow)
execute(val,mod)	GEN	execute specified response (val) in mode mod
growts(ts)	EM	increase ts activation to acquire dominance
incstr(o,t)	GEN	increase activation/strength of object o of type t
learn(val,s,r)	PLTM	use value to adapt rule strength or create new s-r rule
loadgoal(o)	EB	load goal object o
mkbind(goal,tgt)	EB	make bind combining goal and target object
mkchunk(o,type)	EB	make a chunk from object o if possible
mkinst(o,type,loc)	EB	make new instance of object o with type at location loc
mktbr(o,t,mode,loc)	EB	make to-be-recalled instance o of type t at location loc
mkts(ts)	EM	instantiate task set ts
refresh(ts,step)	EB	if ts active, refresh current object (step: start or continue)
rehinit()	PL	initialise or adapt parameter settings for rehearsal
release(o)	EB	deactivate object o
respond(resp)	EB	create the specified response object
retrieve(opt)	GEN	retrieve as specified in opt
revinit()	VSM	initialise/adapt parameter settings for revival
setgams(par,val)	GEN	set general parameter par to value val
setmode(ts,val)	EM	set the mode parameter of ts to value val
setpar(ts,par,val)	EM	set parameter par to value val in ts
unbind(tgt)	EB	unlink and release the bind tgt

Note. The module abbreviations in the second column are: EB (episodic buffer), EM (executive memory), PL (phonological loop), VSM (visuospatial memory), PLTM (procedural LTM), GEN (general).
